# *Culex* mosquitoes in a French Guiana zoo: insights on species diversity, feeding habits, and parasitic associations

**DOI:** 10.1186/s13071-026-07377-2

**Published:** 2026-05-13

**Authors:** Amandine Guidez, Guillaume Lacour, Stanislas Talaga, Romuald Carinci, Pascal Gaborit, Dominique Rousset, Laetitia Bremand, Sourakhata Tirera, Celia Rouges, Linda Duval, Fréderic Ariey, Anne Lavergne, Isabelle Dusfour, Jean-Bernard Duchemin

**Affiliations:** 1 Unité d’Entomologie Médicale, Institut Pasteur de la Guyane, Cayenne, France; 2 Altopictus, Pérols, France; 3 Centre National de Référence des Arbovirus, Laboratoire Associé, Institut Pasteur de la Guyane, Cayenne, France; 4 Laboratoire des Interactions Virus-Hôtes, Institut Pasteur de la Guyane, Cayenne, France; 5 Institut Pasteur, Université Paris Cité, Paris, France; 6 Service de Parasitologie-Mycologie, Hôpital Cochin, Assistance Publique-Hôpitaux de Paris (AP-HP), Paris, France; 7 Département Adaptations du Vivant (AVIV), Molécules de Communication et Adaptation des Microorganismes (MCAM, UMR 7245 CNRS), Muséum National d’Histoire Naturelle, CNRS, CP 52, Paris, France; 8 Inserm U1344, MERIT IRD, Université Paris Cité, Paris, France

**Keywords:** *Culex* female species, Blood meal, Host–mosquito interactions, Haemosporidian parasites, Kinetoplastid parasites, Host feeding habits

## Abstract

**Background:**

French Guiana is home to one of the highest mosquito diversities in the world, with currently 242 species recorded, nearly half of them belonging to the genus *Culex*. These mosquitoes include vectors of human parasites and viruses, some of which are of significant public health concern. Understanding the host preferences of female *Culex* mosquitoes is essential but knowledge is still limited. The Zoo of French Guiana was chosen as an experimental site for its constant availability of diverse captive animals and a potential sentinel role for monitoring infectious diseases.

**Methods:**

Between February 2018 and January 2019, 7892 adult mosquitoes were collected from 42 potential resting sites using manual aspiration. Blood-fed *Culex* accounted for 81% of the total number of blood-fed mosquitoes. Of these, 510 *Culex* female specimens were identified to species level by DNA barcoding, and their blood meals and parasites were analyzed using next-generation sequencing (NGS) technology.

**Results:**

In total, 27 *Culex* species were identified at the zoo, and their blood meals revealed a broad range of vertebrate hosts, with relatively few being human. *Culex eastor* and *Culex vaxus* were the two most common species, feeding mainly on *Tapirus terrestris* and *Iguana iguana*, respectively. In addition, 60% of the mosquito species were infected with blood parasites, either known or novel (haemosporidian and kinetoplastid parasites).

**Conclusions:**

This study provides baseline insights into the diversity, the host associations, and the parasite communities of *Culex* mosquitoes in French Guiana. The zoo appears to be a valuable sentinel site, with mosquitoes feeding on a wide range of vertebrates, providing a foundation for future monitoring and research on mosquito–host–pathogen interactions.

**Graphical Abstract:**

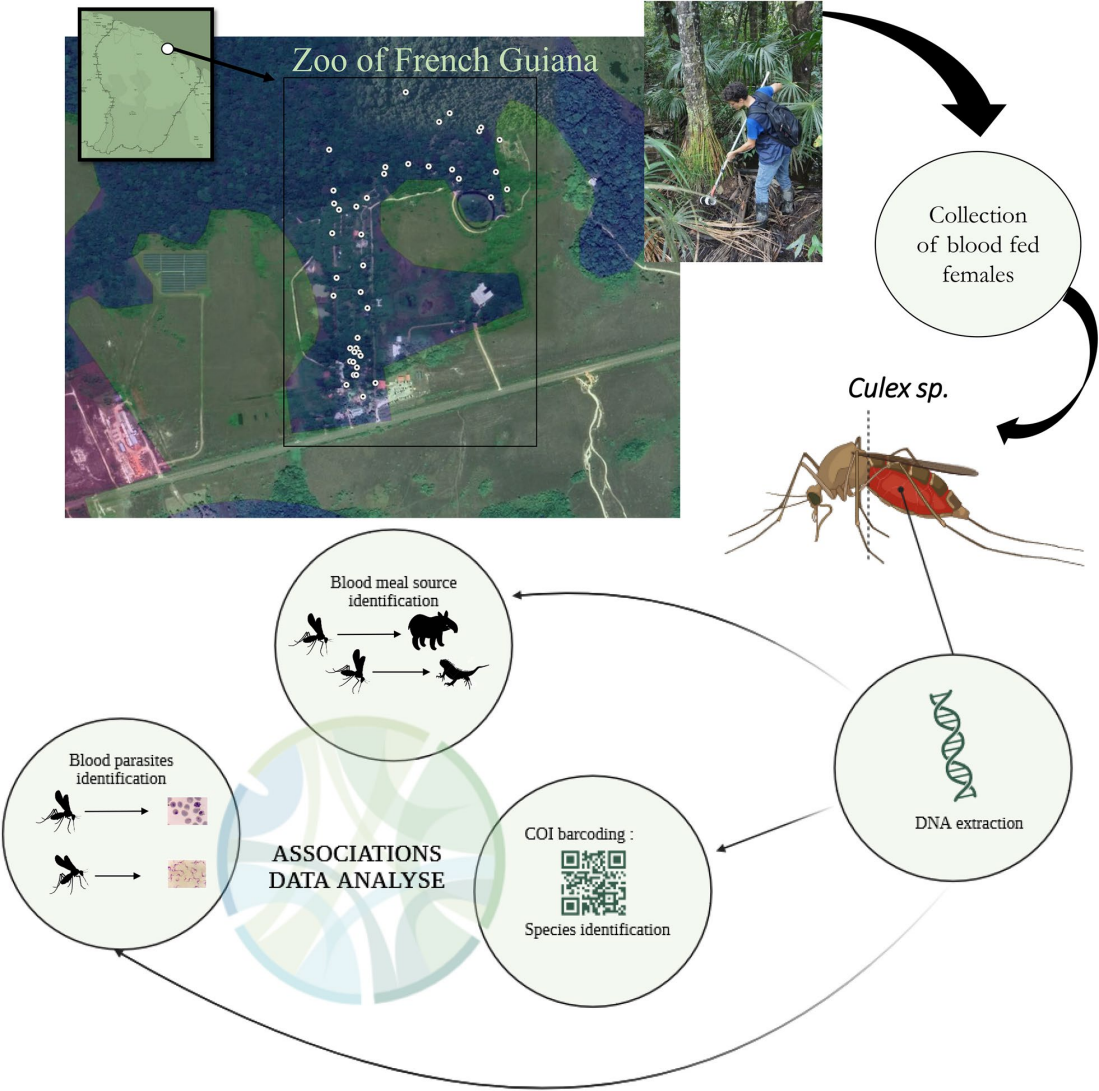

**Supplementary Information:**

The online version contains supplementary material available at 10.1186/s13071-026-07377-2.

## Background

Mosquitoes are hematophagous arthropods that can acquire a pathogen during blood ingestion from an infected host [1]. The choice of blood host is a critical determinant of pathogen exposure, as different hosts harbor distinct types of infectious agents. The coexistence of a host, a vector, and a pathogen can lead to transmission cycles of mosquito-borne pathogens [2]. Studying these cycles is important because 60% of human infections are zoonoses originating from animal reservoirs [3]. Zoos and wildlife parks with captive fauna are excellent sites for studying the ecology and epidemiology of vector-borne pathogens as they combine a complex network of interactions within a limited area [4]. They can have epidemiological consequences for captive and wild animals and humans. Indeed, zoos may harbor vertebrate species from other regions, which increases the risk of introducing foreign pathogens and also makes these animals, with a relatively naive immune systems, vulnerable to local blood-feeding insects and the pathogens they transmit, thus favoring the spillover of pathogens of foreign origin to local fauna or humans. High host heterogeneity and density in zoos may contribute to increased pathogen prevalence, leading to epizootics [5], or, on the contrary, through a dilution effect, to decreased pathogen incidence [6]. For example, monitoring wild crows found dead in the Bronx Zoo in New York led to the discovery of West Nile virus (WNV) as a cause of human morbidity and mortality in the USA in 1999 [7–10]. Diseases caused by blood parasites, can also be prevalent and spread easily in zoos, potentially leading to fatal outcomes for some bird species [11, 12].

The constant availability of host animal species, combined with various breeding sites created within the zoo, makes these habitats attractive to many mosquitoes [13]. Mosquitoes express different degrees of preference toward hosts depending on behavioral, ecological, and physiological factors [1]. Some mosquito species are generalists and show opportunistic feeding behavior, whereas others are specialists and feed preferentially on selected hosts [14]. Species that express strong and inherent host-selection behavior may belong to the most important vectors of infectious diseases among their preys [1]. Understanding host feeding behavior and its variation among mosquito species is crucial for assessing their role in transmission cycles. Blood-fed female mosquitoes provide valuable information about both host use and potential pathogen associations. However, collecting engorged females in the field is often difficult, as they are captured far less frequently than aggressive and unfed individuals. Blood-meal analysis can be complemented by evidence of parasite transmission routes, since many parasites exhibit host specificity and can serve as markers of past feeding events. Investigating these associations offers key insights into blood-source identification and the dynamics of host–parasite–vector interactions.

Covering ~84,000 km^2^ in the northeastern Amazon Basin, French Guiana harbors one of the highest relative mosquito species densities worldwide [16, 17], with 242 recorded species, nearly 45% of which belong to the genus *Culex* (113 species). Mosquitoes of the genus *Culex* play a central role in the transmission of several arboviruses as well as avian malaria, making them a critical target for surveillance [15]. Owing to the difficulties of morphological identification of *Culex* females, specific data on their preferred hosts and the parasites they carry remain scarce in South America.

In this study, we analyzed blood meals from female *Culex* mosquitoes collected over 1 year in a French Guiana zoo that mainly houses local vertebrate species. Our objectives were to assess the diversity of *Culex* species present in the study area, to characterize their host-feeding patterns, and to evaluate the prevalence of haemosporidian and kinetoplastid parasites in their blood meals. Knowledge of blood-meal origins and of the transmission of blood parasites in zoo settings provides insights into the diversity of potential vectors that may affect both wildlife conservation and veterinary and human health.

## Methods

### Study area and mosquito sampling

The Zoo of French Guiana is a 5-hectare zoological garden located in the Macouria municipality, housing 64 animal species in captivity, all originating from the Amazonian region. The zoo occupies a rural zone, surrounded by forest and farmland, and the nearest habitation is over 400 m away. Unlike other zoos that mainly host non-native species, this zoo primarily houses native species and is adjacent to natural areas, leading to frequent interactions with other wild animals.

**Fig. 1:**
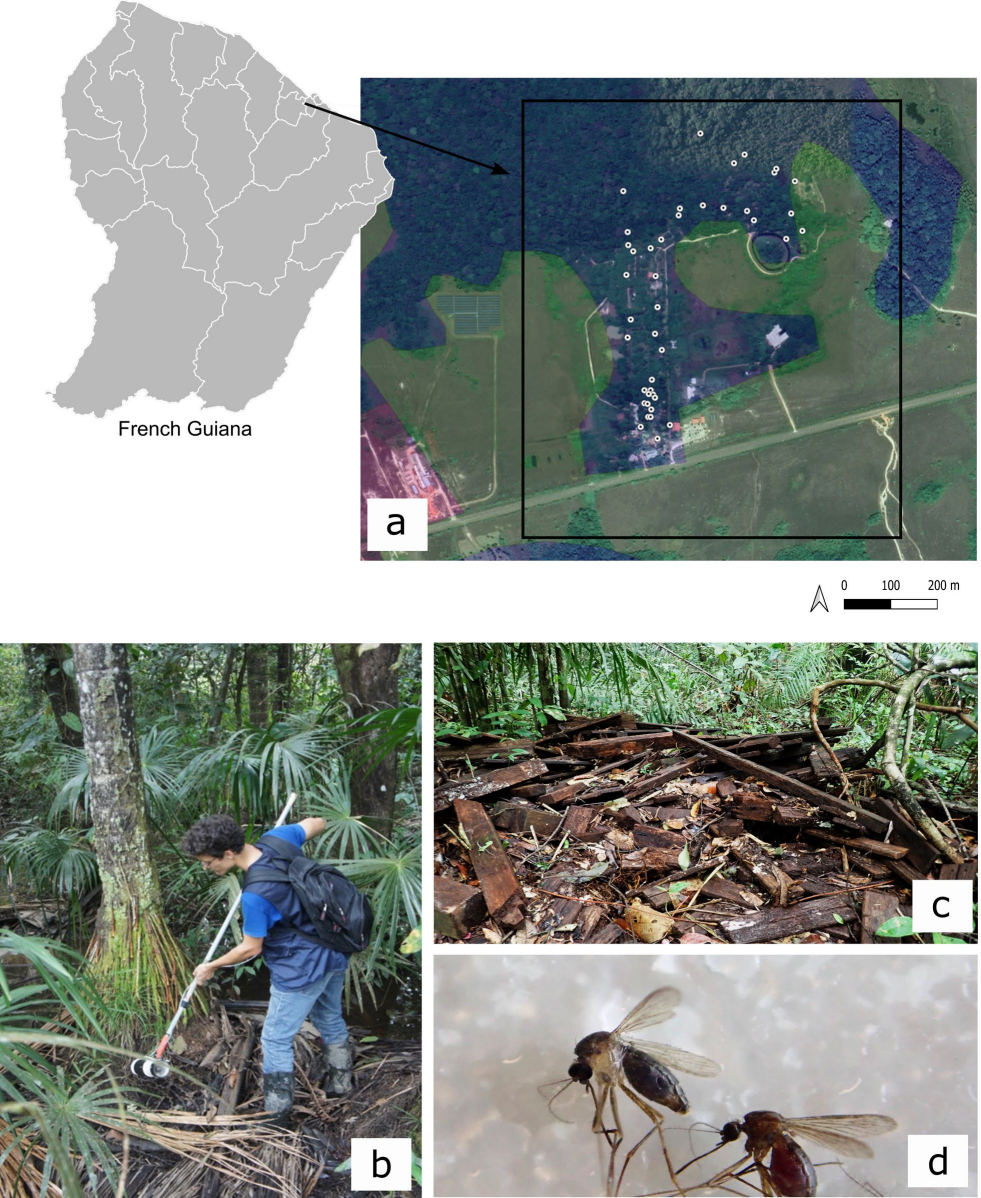
Location of the French Guiana Zoo and resting sites, with illustrative examples of sampling methods, resting sites, and blood-fed female mosquitoes. **a** Map of the zoo with resting sites. **b** Prokopack backpack aspirator in use. **c** Wood piles as resting sites. **d** Blood-fed female mosquitoes

This study was specifically designed to sample resting female mosquitoes. First, we identified natural or preexisting mosquito resting sites in the zoo, such as understory vegetation or wood piles (Fig. 1). The criteria for resting site selection were also related to host proximity, cover, and logistics. Then, 42 potential resting sites distributed throughout the entire zoo were manually aspirated for 90 s during morning capture sessions (from 09:00 to 11:30), using the CDC Backpack Aspirator Mod. 2846, BioQuip, CA, USA, then the Improved Prokopack Aspirator, Mod. 1419, John W. Hock Company, FL, USA (Fig. 1). A total of 35 aspiration sessions were performed from 23 February 2018 to 29 January 2019 (weekly collections from February to July, then one to three sessions per month from August to January). CDC-light traps were tested for 1 month, but manual aspiration was prioritized as more reliable for capturing blood-fed resting mosquitoes in the zoo’s context.

Captured mosquitoes were identified to species level on the basis of morphological characters, using local keys [16–20] whenever possible. Specimens with morphological damage that prevented reliable species identification, as well as females that are particularly difficult—and sometimes impossible—to distinguish morphologically, were only identified to genus level. Males and unfed females were kept aside and pooled by sex, species/genus, date, and sampling location. The average monthly number of *Culex* was computed using the total number of *Culex* captured divided by the number of capture sessions in that month. Blood-fed females were individualized, and classified according to Sella’s stages [21], before storage at −20 °C. A sub-sample of blood-fed females from the dominant genus *Culex* was selected for molecular analyses.

### Molecular identification of engorged *Culex* species

Because females of this genus are difficult to distinguish morphologically, molecular identification was required to obtain reliable species-level data. Abdomens were chosen as providing material for mosquito identification, blood-meal origin, and parasite detection. Dissected abdomens were homogenized in 250 µL of Dulbecco’s modified Eagle medium (DMEM) per sample using sterile plastic pestles or beads with a TissueLyser at 24 Hz for 2 min. The supernatant of the cleared lysate, obtained by centrifugation, was used for DNA extraction. Genomic DNA was then extracted from each *Culex* female using the Macherey–Nagel Tissue DNA Kit, following the manufacturer’s instructions. Extracts were stored at −20 °C. Polymerase chain reaction (PCR) amplification was performed using the primers LCO1490 and HCO2198 [22] to obtain a 758 base pair amplicon of the cytochrome c oxidase subunit I (*COI*) gene [23]. Positive PCR products were sent to Genewiz (https://www.genewiz.com/) for Sanger sequencing. Successful sequences were edited, aligned, and analyzed using MEGA X software and compared with sequences in the Barcode of Life Data System (BOLD). Only sequences with the best match, considering identity (> 97.5%) and best e-score, were retained for analysis.

### Next-generation sequencing (NGS) technologies for identification of blood-meal sources and parasites

Once species identification was established by barcoding, DNA of specimens were pooled (1–10 mosquitoes) by species and used in several PCR amplifications. In order to identify multiple blood meals and to obtain a wide taxonomic coverage, four PCR and one nested-PCR were performed for host identification in female pools and three nested-PCR were performed for parasites screening. All PCR primers are summarized in Additional File 1: Table S1.

PCR products were end-repaired, A-tailed, and ligated to sequencing indexed adapters and used for library construction. Genomic DNA libraries for high-throughput sequencing were prepared using the KAPA HyperPrep Library Preparation Kit (Roche, Switzerland) or the Illumina DNA Prep workflow (Illumina, USA) according to the manufacturer’s recommendations. Libraries were checked for quality and quantity using Qubit for concentration and the Agilent 2100 BioAnalyzer for fragment size. The libraries were sequenced on an Illumina NextSeq 500 instrument using 150-bp paired-end sequencing chemistry on the GENOM’IC platform at the Institut Cochin (Paris, France). A FASTQ file was generated for each pool, containing sequences for vertebrates and, when possible, for parasites.

### Bioinformatic treatment and taxonomic assignment

For identification of host blood-meal species, FASTQ files containing sequences from each pool were aggregated and processed through an automated program to retrieve data using NCBI’s BLASTn tool, which selects sequences on the basis of the highest e-score. All sequences with more than 96% identity were classified at the species level, those with 95–96% identity were classified at the genus level, and the remaining sequences were not retained. Pools not meeting these criteria were also excluded from the analysis.

For parasite screening, after a quality check (FastQC), reads were de novo assembled using SPAdes. We then used NCBI BLAST (blast.ncbi.nlm.nih.gov/) on the contigs longer than 200 base pairs to identify the closest parasite species and retrieve the reference sequence. We used these reference sequences to realign (using BWA) the FASTQ files from each sample. Only sequences showing alignments over 90% coverage compared with the reference sequences were selected for subsequent analysis. Haemosporidian sequences were also screened against MalAvi, a specialized database dedicated to avian haemosporidian parasite sequences [24], to assign species and genetic lineages (https://tavimalara.shinyapps.io/malavi_tables/).

### Data analyses for mosquito–vertebrate associations

Each pool was assigned to a specific mosquito species, although multiple pools could belong to the same species. Results were aggregated by species, with duplicates removed, to ensure that the data accurately reflect the diversity and associations related to each mosquito species.

Hosts of blood meals could be identified to the species or genus level. They were also categorized by host type (generally grouped by order) and by class: Mammalia for all mammals; Aves for birds; Sauropsida for squamates, crocodiles, and turtles; and Amphibia for amphibians.

Mosquito–host associations were evaluated by calculating distances between mosquito species using the Euclidean distance metric. This metric measures the similarity or dissimilarity between species on the basis of their associations with various host categories: mammals, birds, squamates, crocodiles, turtles, and amphibians. Hierarchical clustering was performed on the normalized data matrix, which involved computing the distance matrix and constructing a hierarchy of clusters by progressively merging the closest pairs of clusters. The complete linkage method was used, which merges clusters on the basis of the maximum distance between any two points within the clusters. The resulting dendrogram visually groups species into clusters according to their similarities.

### Parasite analysis

To assess the phylogenetic position of parasites belonging to the haemosporidian species, a phylogenetic tree was calculated on the basis of sequences available in the GenBank database (https://www.ncbi.nlm.nih.gov/) and using sequences obtained in this study. The maximum likelihood (ML) method in MEGA X was used [25] with the best substitution model for all alignments (GTR + invariant sites + gamma distribution) and bootstrap analysis using 1000 replicates per tree. In a similar manner, an ML phylogenetic tree was used to examine the relationships between sequences of kinetoplastids, on the basis of sequences available in the GenBank database and sequences obtained in this study. The best substitution model for all alignments (Tamura Nei + gamma distribution) was applied, and bootstrap analysis was performed using 1000 replicates per tree. The infection rate of mosquitoes was estimated using the minimum infection rate (MIR), calculated with the formula: [(number of positive mosquito pools/total number of mosquitoes in pools tested) × 1000].

## Results

### Mosquito collection and blood-fed *Culex*

In total, 7892 adult mosquitoes were collected from 42 resting sites, comprising 4601 females and 3291 males. Among the females, 31% (1442) were blood-fed, with 65% of them being at Sella stage 2. The *Culex* genus was found to be the most abundant, accounting for 80.9% of the total blood-fed females (1196/1442), followed by the *Coquillettidia* genus at 9.8% (139/1442), while the other genera were only represented by a few specimens (Additional file 2: Fig. S1). The average number of blood-fed *Culex* varied from 35 to 1196 depending on the month, with peaks in mosquito abundance in July and December‒January (Additional file 2: Fig. S1).

### *Culex* species identification and sample collection

A total of 1196 blood-fed females of the genus *Culex* were collected. Given the difficulty of accurately identifying *Culex* species based solely on morphology, a random selection of 632 specimens (54%) was analyzed to provide an overall view of the species present on the site throughout the year (Additional file 3: Fig. S2). To account for temporal variation, the number of specimens was balanced by month, resulting in random monthly subsamples of the collected *Culex* females. These subsamples were analyzed using *COI* barcoding PCR for molecular species identification.

A total of 510 samples (81%) was identified to species level with more than 96% identity confidence (Additional file 3: Fig. S2). This subsampling allowed the identification of 27 *Culex* species, including five predominant species: *Culex eastor*, *Culex vaxus*, *Culex spissipes*, *Culex dunni*, and *Culex pleuristriatus* (Additional file 3: Fig. S2). Detailed information on collection and identification can be found in Additional file 4: Table S2.

### Blood-meal analyses

#### Comparison of zoo vertebrates and mosquito blood-meal hosts

**Fig. 2:**
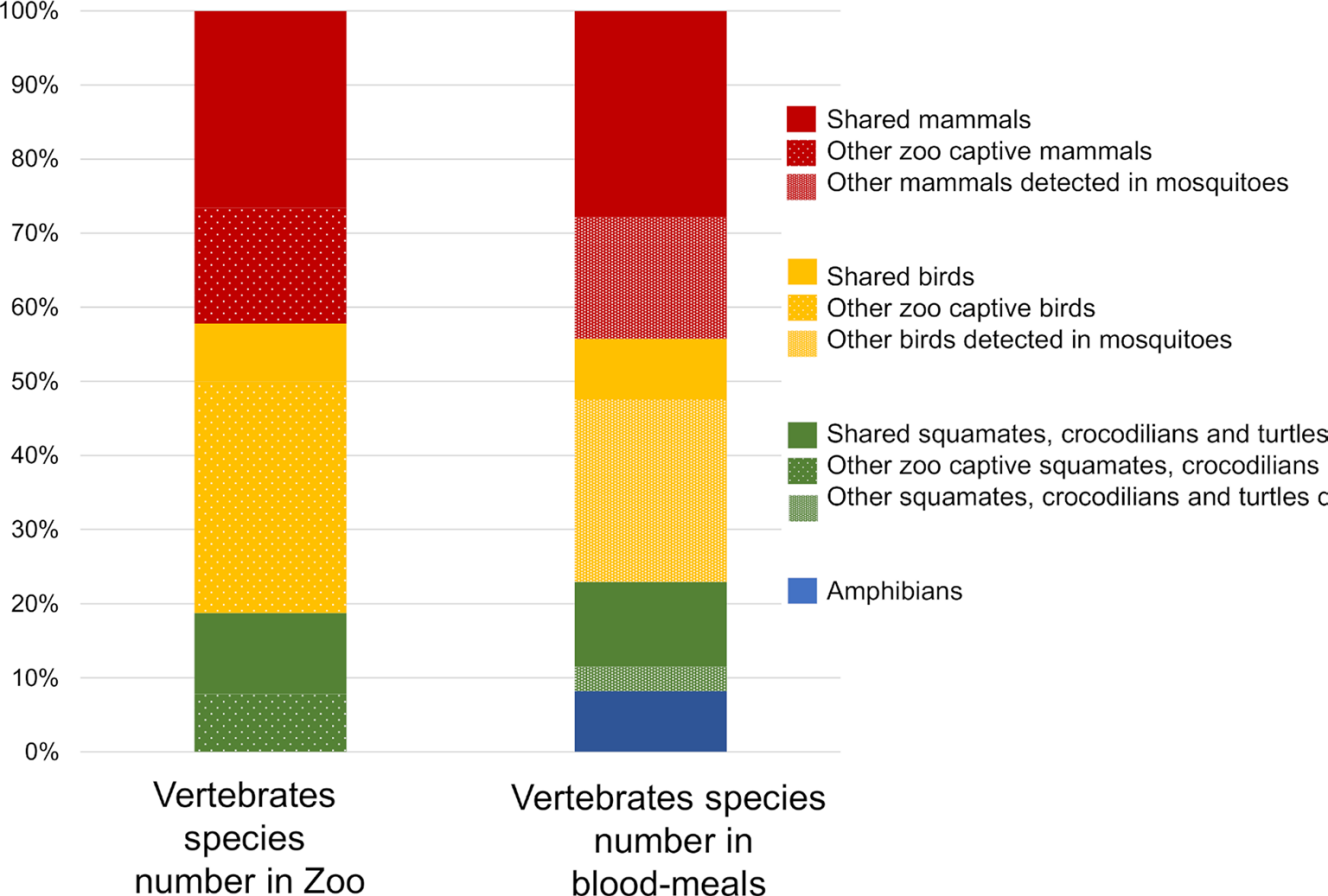
Comparative analysis of zoo vertebrate species representation and *Culex* mosquito blood-meal hosts. Comparison of the proportion of zoo species (%) at the zoo site and in the blood-meal hosts of *Culex* mosquitoes in the study. Mammals are shown in red, birds in yellow, squamates/crocodiles/turtles in green, and amphibians in blue. The proportion of species found in zoo enclosures is represented by widely spaced dots, and the proportion of species detected only in mosquito blood meals is indicated by closely spaced dots for each group

At the zoo site, 27 species of mammals, 25 species of birds, 5 species of squamates, 4 species of turtles, and 3 species of crocodiles were present in captivity. The known vertebrate diversity of the site, based on captive animals, was compared with the vertebrates detected in the blood-meals of engorged female mosquitoes (Fig. 2). Mosquito blood-meals detected 63% of captive mammal species (17/27), 20% of captive bird species (5/25), and 58% of captive squamate, turtle, and crocodile species (7/12). In addition, five amphibian species were detected in mosquito blood meals.

#### Mosquito–vertebrate associations

**Fig. 3:**
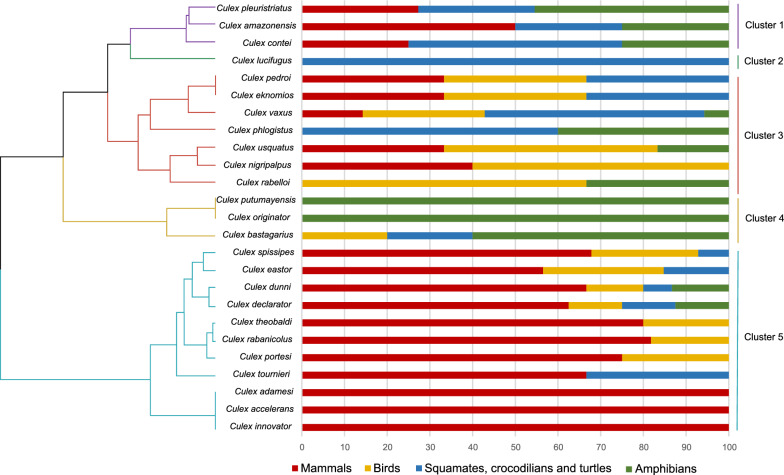
Hierarchical clustering of mosquito species based on blood-meal associations

The hierarchical clustering analysis, as visualized in the dendrogram in Fig. 3, identified five distinct clusters of mosquito species on the basis of their blood-meal abundance across various host categories: mammals, birds, squamates, crocodiles, turtles, and amphibians. Cluster 1 comprises species that exhibit diverse feeding behaviors with relatively balanced preferences across multiple host categories. Cluster 2 is characterized by species showing a strong association with squamates, crocodiles, and turtles. With the exception of *Cx. phlogistus*, cluster 3 includes species that primarily feed on birds, while also exhibiting variable but sometimes substantial associations with mammals and other host categories. Cluster 4 consists of species with a notable preference for amphibians, together with varying degrees of association with other hosts. Finally, cluster 5 includes 11 species that feed predominantly on mammals with minimal associations with other host categories. These clusters provide an overview of the distinct feeding strategies and host preferences among mosquito species.

*Culex* mosquito species exploit a wide variety of hosts across multiple taxonomic groups, including mammals, birds, squamates, amphibians, and even crocodiles and turtles. The number of blood meals associated with each mosquito species from specific host taxa is indicated in Additional file 5: Table S3. *Culex eastor*, *Cx. vaxus*, and *Cx. spissipes* show the highest diversity in mammal hosts. *Tapirus terrestris* (South American tapir) is a prominent mammalian host, particularly for *Cx. eastor* (11 meals). Other significant mammal hosts include *Dasyprocta leporina* (red-rumped agouti), *Choloepus didactylus* (Linnaeus’s two-toed sloth), and *Philander opossum* (opossum), with varying frequencies across several *Culex* species. The data show that reptilian hosts, particularly *Iguana iguana* (green iguana), are a significant source of blood meals for certain *Culex* mosquito species. *Culex vaxus* appears to have a strong preference for *Iguana iguana*, with 11 recorded meals, while *Cx. eastor* fed on this species only once. Amphibians, particularly *Osteocephalus taurinus* (Giant broad-headed treefrog) and *Scinax ruber* (red-snouted treefrog), are more frequently targeted by mosquitoes such as *Cx. pleuristriastus* and *Cx. dunni*. Though less frequently targeted, crocodiles and turtles are part of the diet of a few mosquito species.

### Molecular characterization and phylogenetic relationships of parasites

#### Haemosporidian parasites

The database analysis revealed 15 pools positive for the presence of haemosporidian parasites from seven mosquito species: *Cx. dunni* (1 pool), *Cx. eastor* (4 pools), *Cx. phlogistus* (1 pool), *Cx. pleuristratus* (1 pool), *Cx. spissipes* (3 pools), *Cx. usquatus* (1 pool), and *Cx. vaxus* (4 pools). The haemosporidian sequences obtained in this study were deposited in GenBank (accession numbers: PX096609–PX096616 and PX113786–PX113794). Molecular analysis of *cytb* sequences of the pools identified seven distinct sequences, all corresponding to *Plasmodium* parasites. More than half of the positive pools (8/15, 53%) contained *Plasmodium floridense*, likely indicating a lizard blood meal, as lizards are the primary hosts of this parasite. Other lizard *Plasmodium* species detected include *Plasmodium kentropyx* and *Plasmodium carmelinoi*. Avian *Plasmodium* species were also identified: *Plasmodium nucleophilum*, *Plasmodium tejerai*, and two additional sequences were related to HMA-2012 *Plasmodium* isolates (TUOLI01 lineage in MalAvi). A summary of mosquito species, associated pools, blood-meal detection, and parasites can be found in Additional file 6: Table S4.

**Fig. 4:**
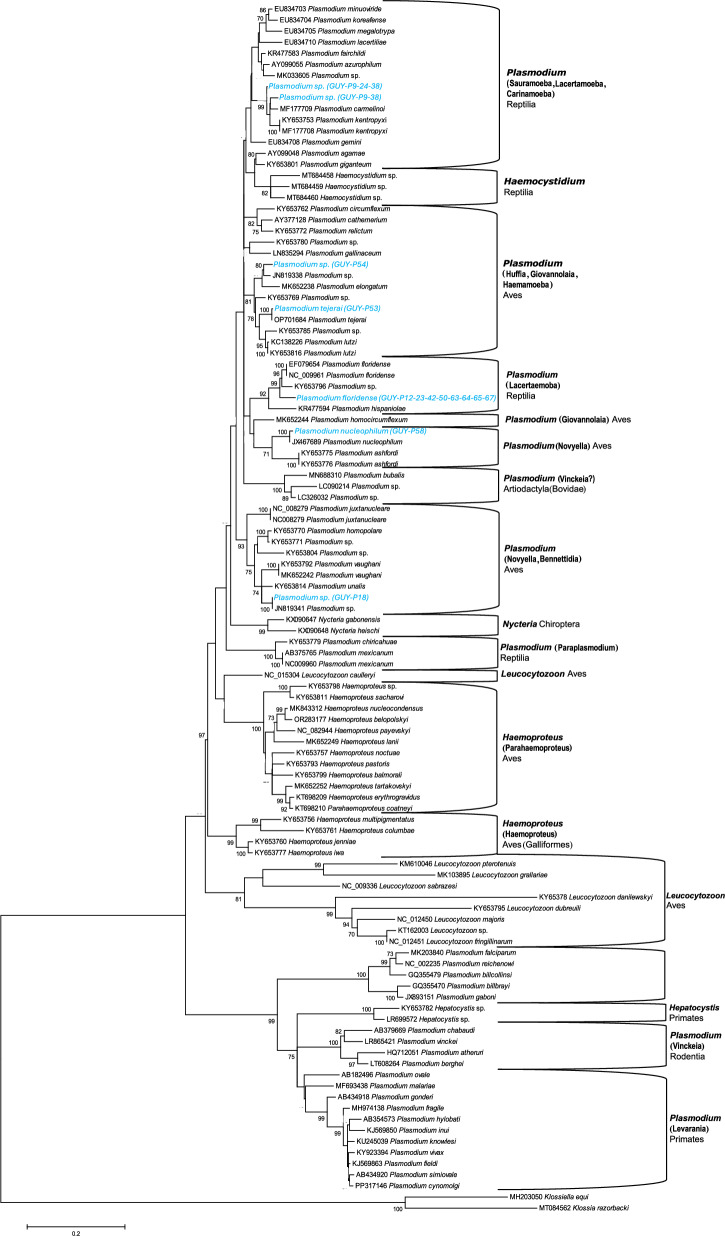
Phylogenetic relationships of haemoparasites. Analysis was inferred by using the maximum likelihood method and general time reversible + I + G model. Analysis involved 109 nucleotide sequences conducted in MEGA X. Sequences obtained in this study are presented in blue. Numbers at nodes correspond to bootstrap values (> 70) using 1000 replicates

The phylogenetic analysis (Fig. 4) supported the association of parasites with birds and sauropsids. Within the birds, four samples shared sequences with *Plasmodium tejerai*, *Plasmodium nucleophilum*, and two *Plasmodium* species, with moderate-to-strong bootstrap support (80–100). For the sauropsids, eight samples clustered with *Plasmodium floridense* sp., also with high support (99), while three samples grouped with *Plasmodium kentropyxi* and *Plasmodium carmelinoi*, although these were not supported by bootstrap analysis.

#### Kinetoplastid parasites

We identified 28 mosquito pools containing kinetoplastids across 16 mosquito species. Molecular sequence analysis of these pools revealed ten distinct *Trypanosoma* species, six *Paratrypanosoma*/*Trypanomatidae* species, one *Endotrypanum* species, one *Neobodo* species, and one *Parabodo* free-living species. All sequences were deposited in GenBank (accession numbers: PV939845–PV939863).

**Fig. 5:**
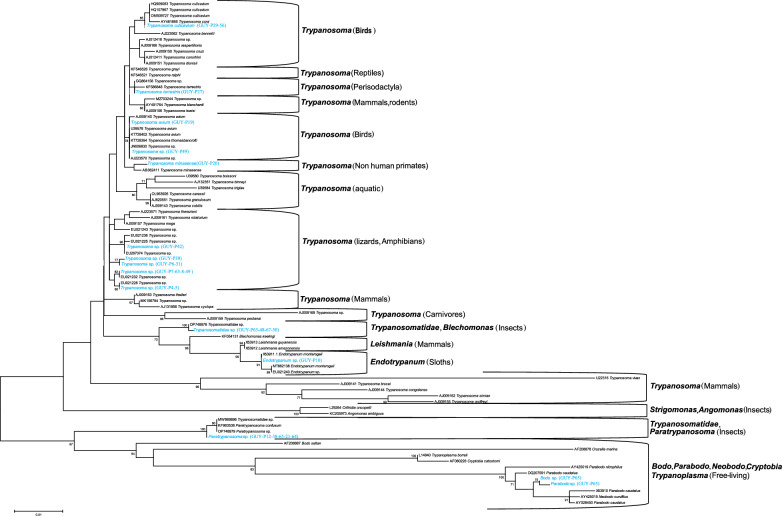
Phylogenetic relationships of kinetoplastids. Analysis was inferred by using the maximum likelihood method and Tamura Nei model. Analysis involved 85 nucleotide sequences conducted in MEGA X. Sequences obtained in this study are highlighted in blue. Numbers at nodes correspond to bootstrap values (> 70) using 1000 replicates

The phylogenetic analysis (Fig. 5) revealed that two samples clustered with *Trypanosoma culicavium* associated with birds, with a high bootstrap value (99%). Two additional samples were also associated with bird *Trypanosoma*, clustering with *Trypanosoma avium* sequences. In addition, a single mosquito pool in the entire dataset contained *Trypanosoma terrestris*. This case was associated with a tapir (*Tapirus terrestris*)-derived blood meal, and the corresponding sequence clustered strongly with *Trypanosoma terrestris* with robust bootstrap support (99%). Another sample pool was associated with *Trypanosoma minasense* with moderate bootstrap support (82%), a parasite of nonhuman primates, whose transmission cycle is still unknown [26]. In addition, four mosquito pools clustered with *Trypanosoma* parasites known to infect lizards and amphibians according to literature, although one sample grouped with the clade but did not closely cluster with any known species. For *Paratrypanosoma* species, four pools clustered strongly with *Paratrypanosoma confusum*, while five samples clustered with an unidentified *Trypanosomatidae* species. An *Endotrypanum* parasite, commonly found in sloths [27], was detected in one mosquito pool with moderate bootstrap support (85%). This finding was corroborated by the presence of sloth DNA in the blood meal of the same pool. Lastly, two parasite species associated with *Neobodo curvifilus* and *Parabodo caudatus* were found to cluster together in another pool.

#### Associations between *Culex* species, hosts, and parasites

The diversity of host species found in *Culex* species pools, along with the detection of specific pathogens (*Plasmodium* and kinetoplastids), is summarized in Additional file 7: Table S5. *Culex eastor* and *Cx. vaxus*, which had the highest number of total samples in pools (113 and 101, respectively), exhibited the most diverse range of vertebrate hosts, including mammals, sauropsids (birds, crocodiles, squamates, turtles), and amphibians. Human blood was rarely detected, with *Cx. vaxus* being the species most frequently associated with human blood. Several mosquito species were found to be associated with the presence of *Plasmodium* or kinetoplastids in their blood meals, indicating either prior blood-meal acquisition or their potential as vectors for these pathogens.

## Discussion

### High biodiversity in the Zoo of French Guiana

Mosquito communities collected in the zoo were dominated by the genus *Culex*. This pattern is consistent with both the environmental context and the sampling strategy. We used aspirators, which are known to be particularly efficient for collecting resting mosquitoes, including blood-fed females, in outdoor resting sites [28, 29]. We also used CDC light traps for a short period, which are known to be particularly attractive to *Culex* females [30]. *Anopheles* species can nevertheless be locally abundant in similar environments, as illustrated by *Anopheles braziliensis*, which was previously recorded in high numbers at the zoo using mosquito magnet traps (MMt) between February and April 2018 (18.5% of captures with MMt versus 0.4% using aspirators; personal data). However, aspirators are known to collect very few *Anopheles* when used on outdoor resting sites [25]. In contrast, forest and savannah habitats typically harbor low abundances of *Aedes* mosquitoes [31].

Our study focused on a subset of blood-fed female mosquitoes collected over a 1-year period, allowing the characterization of *Culex* species diversity at the site. A total of 27 *Culex* species were identified, representing approximately one-quarter of the species currently known in French Guiana. This highlights the zoo as a highly favorable environment for *Culex* mosquitoes for which knowledge gaps persist concerning their ecology and trophic preferences.

From a vertebrate species perspective, the site hosts 64 captive species, to which 32 other vertebrate species were identified through mosquito blood meals. This brings the total number of documented host vertebrate species interacting with mosquitoes at the site to 96 species. Analysis of blood meals revealed that *Culex* mosquitoes fed predominantly on mammals, followed closely by birds, and then by sauropsids and amphibians. Among the various hosts, the lizard *Iguana iguana* (green iguana) was the most frequently detected across all mosquito species, with *Cx. vaxus* showing a strong preference, feeding on this species 61% of the time. *Tapirus terrestris* (South American tapir) was the mammal most commonly fed upon by *Cx. eastor*. In addition, the sloth *Choloepus didactylus* (Linnaeus’s two-toed sloth) and the amphibian *Osteocephalus taurinus* (giant broad-headed treefrog) were also frequently found in the blood meals of *Culex* species.

### Two predominant *Culex* species

Among the mosquito assemblage, *Cx. eastor* and *Cx. vaxus* were the two predominant species and both species were consistently present throughout the year. Because these species can be difficult to identify morphologically and are easily confused with closely related species [20], information on them remains scarce.

*Culex vaxus* exhibits behavioral traits commonly shared with forest mosquitoes, as it tends to poorly adapt to environments where forest cover is reduced [32]. In the sampled and identified females, *Cx. vaxus* fed on humans four times more frequently than *Cx. eastor*. Reports of dengue virus serotype 2 (DENV-2; family *Flaviviridae*) detection in *Cx. vaxus* pools in Brazil [32] support its preference toward human hosts. Although known for its eclectic trophic behavior [32, 33], at the zoo, *Cx. vaxus* displayed a strong feeding preference for the lizard *Iguana iguana*. In any case, the data obtained in this study provide additional information on the ecological flexibility of this understudied *Culex* species.

### Low interactions with humans

Human blood meals were detected mainly in *Cx. vaxus* but were also observed in *Cx. eastor*, *Cx. theobaldi*, *Cx. pleuristriatus*, *Cx. contei*, and *Cx. pedroi*, always at low frequencies and often associated with nonhuman hosts. Human presence at the zoo is restricted to daytime hours, with no visitor access during the evening and night, a factor that is particularly relevant given that most *Culex* species bite at night or at dusk. Engorged mosquitoes were collected in the morning, likely after nocturnal feeding events, further indicating limited opportunities for human–mosquito contact at the site. This interpretation is consistent with the absence of human blood meals in several *Culex* species for which human feeding has been reported in other ecological contexts, including *Cx. nigripalpus* [34]. Nevertheless, shifts in trophic preferences may also reflect the specific ecological context represented by the zoo. In any case, the low rate of human biting offers a useful framework for exploring wildlife-associated transmission cycles and should be considered when interpreting the diversity and origin of the detected parasite lineages.

### Parasites as molecular markers of feeding habits of mosquitoes

Invertebrate-derived DNA (iDNA) has recently emerged [35] as a method to analyze dipteran blood meals to detect hosts and screen for vertebrates [35–39]. While iDNA surveys have successfully identified host DNA [35, 36, 39–41] capturing blood-fed females is challenging owing to rapid blood digestion. Parasites detected within mosquitoes can therefore act as indirect molecular markers of previous feeding events, particularly when parasites show strong host specificity. In this study, *Plasmodium floridense* was detected in *Cx. eastor* in the absence of detectable lizard blood, suggesting parasite persistence from a previous blood meal. Given the difficulty in capturing many blood-fed females, this technique is already used on non-fed females, revealing past blood meals [35–37, 41]. However, despite advances in molecular screening, the database of parasites in wild vertebrates remains incomplete, emphasizing the need to improve baseline knowledge of host–parasite associations before fully exploiting this approach.

### Parasite DNA detection in *Culex* mosquito species

Haemoparasites (notably *Plasmodium* spp.) and kinetoplastid lineages (including *Trypanosoma* spp.) are widely distributed vector-borne parasites infecting reptiles, birds, and mammals. Their detection in mosquitoes provides insight into parasite circulation as well as mosquito feeding habits and host contact networks. Molecular screening revealed a diverse assemblage of these parasites in *Culex* mosquitoes collected at the zoo. More than half of the *Culex* species tested on site harbored parasite DNA, with *Plasmodium* DNA detected in 7 mosquito species (15 pools) and trypanosomatidae DNA (including *Trypanosoma*, *Paratrypanosoma*, and *Endotrypanum*) in 15 mosquito species (25 pools).

The detection of parasite lineages known to infect reptiles, birds, and mammals often matched the vertebrate hosts identified in corresponding blood meals, supporting the biological coherence of the molecular signal. For instance, reptilian *Plasmodium kentropyxi*, reported from the teiid lizard *Kentropyx calcarata* [42], was detected in pools of *Cx. eastor* containing blood meals from this host. Similarly, avian *Plasmodium* sequences (*Plasmodium tejerai*, *Plasmodium nucleophilum*) were detected in association with bird-derived blood meals, including from *Cx. usquatus* and *Cx. spissipes*, confirming the *Plasmodium nucleophilum* previously reported infection of toco toucans at the zoo [12]. Among trypanosomatidae, *Trypanosoma culicavium* and *Trypanosoma avium* were detected in mosquito pools containing avian blood meals, while *Trypanosoma terrestris* was identified alongside its known mammalian host, *Tapirus terrestris* (South American tapir) [43].

In contrast, some parasites were detected in mosquito pools in which the expected vertebrate host was not identified in the blood meal. For example, *Plasmodium carmelinoi* and *Trypanosoma minasense* were found in pools lacking reptile or primate blood sources, respectively. These detections probably reflect infections acquired during previous blood meals that persisted across gonotrophic cycles.

In addition to known parasite species, several mosquito pools contained parasite DNA that could not be confidently assigned to any described parasite species. Phylogenetic analyses revealed that some of these sequences clustered with avian-associated *Plasmodium* species, suggesting that birds are the most likely vertebrate hosts. In one case, an unidentified *Plasmodium* sequence detected in a *Cx. eastor* pool originated from a mosquito that had fed on a passerine bird and showed a 100% sequence identity with an unidentified *Plasmodium* previously reported from a passerine, further supporting this host association. Other unidentified lineages detected in *Cx. spissipes* and *Cx. eastor* lacked a corresponding vertebrate host in the blood meal. Unidentified kinetoplastid lineages were also detected, including several *Paratrypanosoma* sequences found in multiple *Culex* species. These lineages clustered within the *Paratrypanosoma* genus on the basis of phylogenetic analyses. However, no definitive host associations could be established for these parasites. Overall, the presence of unidentified parasite lineages indicates a diversity not fully resolved in current reference databases.

In four mosquito pools, co-occurring parasites were detected. Three of these pools contained up to six mosquitoes, making it difficult to determine whether the co-infections originated from multiple mosquitoes. In contrast, one pool consisting of a single *Cx. eastor* revealed a high diversity of host blood meals, including a *Myotis* bat, a passerine bird, and a *Kentropyx* lizard with a co-infection with *Plasmodium kentropyxi* and *Plasmodium carmelinoi*, both lizard parasites. *Plasmodium kentropyxi* is specific to the teiid lizard *Kentropyx calcarata*, a species found in French Guiana and confirmed in our blood meal analysis, validating the accuracy of this result. These findings again highlight the complexity of host–parasite exposure in this ecosystem, indicating that mosquitoes may acquire parasites from different hosts over successive feeding events.

It is important to note that in order to determine whether a mosquito species is a competent vector for a parasite, it is necessary to detect the infectious stages of the parasites in the insect’s salivary glands [44]. This would require insect dissection and slide preparation [45], or vector competence laboratory studies [46]. Our results are based solely on the molecular analysis of female abdomens; the exclusion of salivary glands does not allow us to determine the vector status of these mosquitoes. Moreover, the focus of our study on blood-fed females, by increasing our chances of finding blood parasites, does not allow these parasites to have completed their full life cycle within the mosquito host to confirm such parasite–host associations.

### Zoos as sentinels of infectious diseases monitoring

Although zoos primarily play a key role in conservation by engaging visitors and the community, they can also serve as sentinel sites for monitoring infectious diseases. The environment of the French Guiana Zoo, with its diverse animal species, including ones that may come into contact with the surrounding wildlife, could be a potential source of infectious diseases. Over the past few decades, captive and free-ranging animals have often been used as sentinels for monitoring arbovirus infections [47, 48]. Following the WNV outbreak in New York City in 1999, monitoring of animals in the zoological park of the city showed that 70% of ill birds died of WNV [7]. Studies focusing on the potential of zoos as sentinel sites for pathogen detection are increasing and are well justified. For instance, research on zoo animals in Austria, Switzerland, and Hungary led to the detection of Usutu virus (USUV) in birds [49, 50]. Similarly, in a Brazilian zoo, a study on trypanosomatid transmission revealed a high frequency of infection with *Trypanosoma cruzi* and *Leishmania* spp. among captive wild mammals, likely owing to local vector-borne transmission.

In this study, we provide information on parasite diversity, focusing exclusively on *Culex* vector species. This approach could be expanded to include the screening of viruses or testing other genera and blood-sucking species as well as testing zoo captive animals. Understanding which species contribute to and lead to disease outbreaks can help us better manage these threats. The follow-up of vertebrate and vector communities in zoos could play a crucial role in the early detection of emerging pathogens and their prevention in the future. This underscores the importance of long-term monitoring of sentinel animal sites for surveillance but also to provide knowledge about local vector communities and their associations with wild animals.

## Conclusions

In this study, we expanded the list of potential *Culex* vectors of parasites. Further research would be needed to clarify the role of mosquitoes in the life cycle of these highlighted parasites. Blood-fed females and molecular DNA identification and monitoring should be encouraged to improve knowledge of the evolutionary and ecological relationships between the highly diverse communities of vertebrates, parasites, and vectors present in the Amazonian region. Zoo settings represent strategic locations at the human–animal interface, providing valuable information on pathogen prevalence and transmission while serving as early warning systems for disease emergence.

## Supplementary Information

Additional file 1 (DOCX 17 KB)

Additional file 2 (PDF 591 KB)

Additional file 3 (PNG 548 KB)

Additional file 4 (DOCX 72 KB)

Additional file 5 (DOCX 27 KB)

Additional file 6 (PDF 43 KB)

Additional file 7 (DOCX 16 KB)

## Data Availability

All sequences of parasites are available on NCBI. Data supporting the main conclusions of this study are included in the manuscript.
