# Supplementary material for: Culex mosquitoes in a French Guiana zoo: insights on species diversity, feeding habits, and parasitic associations
Source: Parasit Vectors. 2026 May 13;19:274. doi: 10.1186/s13071-026-07377-2 (PMC13339560; doi:10.1186/s13071-026-07377-2)
Supplement: Supplementary file 1 — Additional file 1 (DOCX 17 KB) [file 13071_2026_7377_MOESM1_ESM.docx]

| **Screening host blood meal sources** | | |  |  |  |
| --- | --- | --- | --- | --- | --- |
| **Gene name** | **Primer names** |  | **Primer Sequences (5'-3')** | **Product size (pb)** | **Reference** |
| *Cyt b* | Cyt_b_F | Forward | GAGGMCAAATATCATTCTGAGG | 457 | *towsen et al, 2018* |
|  | Cyt_b_R | Reverse | TAGGGCVAGGACTCCTCCTAGT |  |  |
|  |  |  |  |  |  |
| *COI* | COI_short_F | Forward | GCAGGAACAGGWTGAACCG | 330 |  |
|  | COI_long_R | Reverse | AAGAATCAGAATARGTGTTG |  |  |
|  | Mod_RepCOI_F | Forward | TNTTYTCMACYAACCACAAAG A | 244 | *Reeves, 2018* |
|  | VertCOI_7216_R | Reverse | CARAAGCTYATGTTRTTYATDCG |  |  |
|  | VertCOI_7194_F | Forward | CGMATRAAYAAYATRAGCTTCTGAY | 395 |  |
|  | Mod_RepCOI_R | Reverse | TTCDGGRTGNCCRAARAATCA |  |  |
|  |  |  |  |  |  |
|  | **PCR 1 :** |  |  |  | *Kocher, 2017* |
| *12S* | Mam12S-340-F | Forward | CCACCGCGGTCATACGATT | 340 |  |
|  | Mam12S-340-R | Reverse | GATGGCGGTATATAGACTG |  |  |
|  | **Nested-PCR 2** |  |  |  |  |
|  | 12S-V5-F | Forward | TAGAACAGGCTCCTCTAG | 100 |  |
|  | 12S-V5-R | Reverse | TTAGATACCCCACTATGC |  |  |
| **Screening blood parasites** | |  |  |  |  |
| **Hemoparasites** |  |  |  |  |  |
| *Cyt b* | **PCR 1 :** |  |  |  | *Amplification through 40 cycles with 30 secondes at 95°C, 30 secondes at 55°C, and one minute at 72°C, followed by a final extension step at 72°C for 10 minutes* |
|  | PLAS1 | Forward | GAGAATTATGGAGTGGATGGTG | 816 |  |
|  | PLAS2a | Reverse | GTGGTAATTGACATCCWATCC |  |  |
|  | **Nested-PCR 2** |  |  |  |  |
|  | PLAS3 | Forward | GGTGTTTYAGATAYATGCAYGC | 787 |  |
|  | PLAS4 | Reverse | CATCCWATCCATARTAWAGCATAG |  |  |
| **kinetoplastids** |  |  |  |  |  |
| *18SrRNA* | **PCR 1 :** |  |  |  | *Amplification through 40 cycles with 30 secondes at 95°C, 45 secondes at 52°C, and two minutes at 72°C, followed by a final extension step at 72°C for 10 minutes* |
|  | SLF | Forward | GCTTGTTTCAAGGACTWAGC | 2260 |  |
|  | S762 | Reverse | GACTTTTGCTTCCTCTAWTG |  |  |
|  | **Nested-PCR 2.1** |  |  |  |  |
|  | S823 | Forward | CGAACAACTGCCCTATCAGC | 973 |  |
|  | S662 | Reverse | GACTACAAYGGTCTCTAATC |  |  |
|  | **Nested-PCR 2.1** |  |  |  |  |
|  | S825 | Forward | ACCGTTTCGGCTTTTGTTGG | 1061 |  |
|  | SLIR | Reverse | ACATTGTAGTGCGCGTGTC |  |  |
|  |  |  |  |  |  |
