# Supplementary material for: Culex mosquitoes in a French Guiana zoo: insights on species diversity, feeding habits, and parasitic associations
Source: Parasit Vectors. 2026 May 13;19:274. doi: 10.1186/s13071-026-07377-2 (PMC13339560; doi:10.1186/s13071-026-07377-2)
Supplement: Supplementary file 2 — Additional file 2 (PDF 591 KB) [file 13071_2026_7377_MOESM2_ESM.pdf]

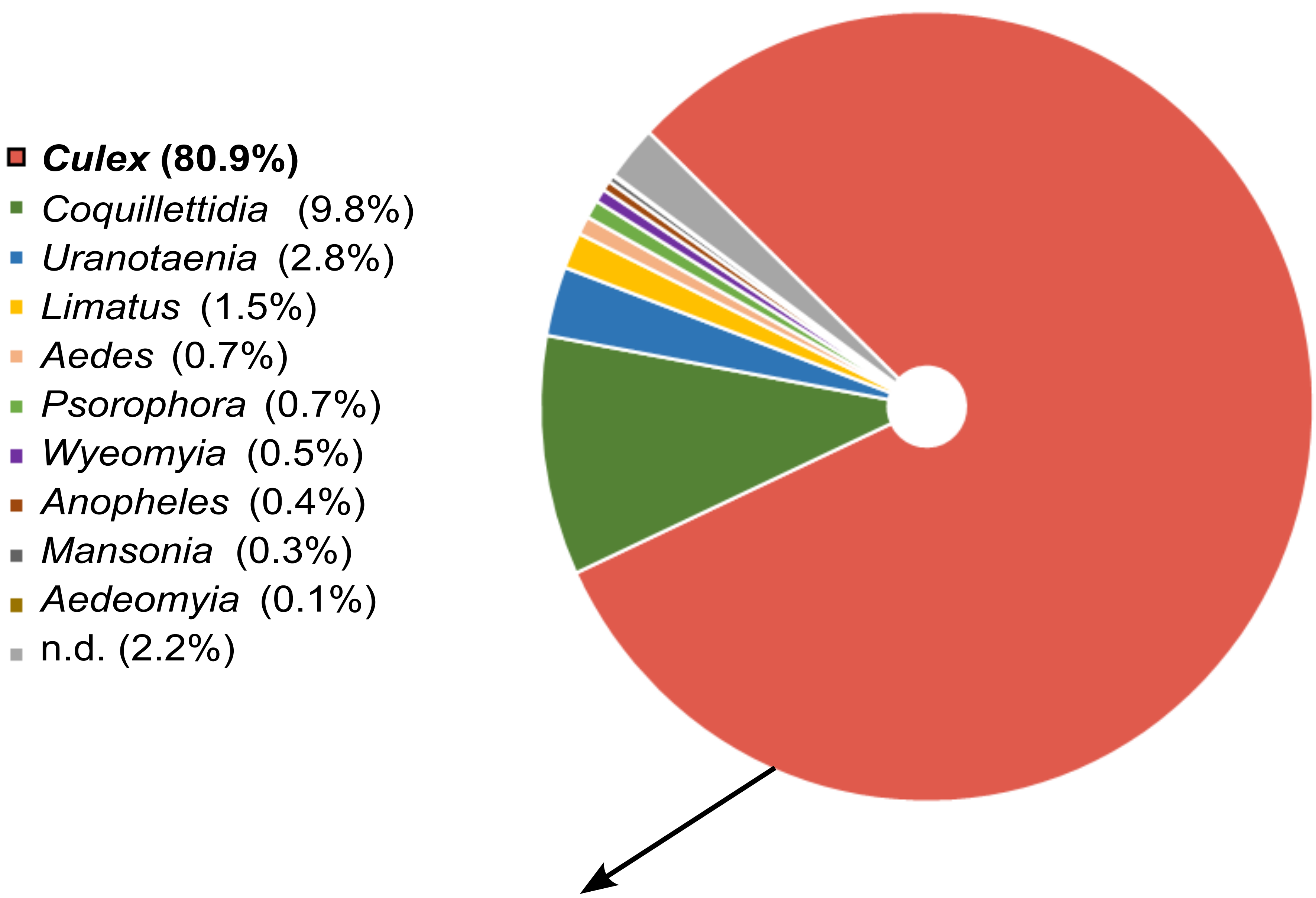

Selection of *Culex* females mosquitoes

Month	Year	Capture session number	Total *Culex* mosquitoes	Males	Non blood-fed females	Blood-fed females
February (Feb)	2018	2	**85**	9	15	61
March (Mar)	2018	5	**174**	54	41	79
April (Apr)	2018	5	**408**	130	112	166
May (May)	2018	4	**394**	154	156	84
June (Jun)	2018	6	**588**	282	117	189
July (Jul)	2018	4	**1042**	331	338	373
August (Aug)	2018	1	**73**	26	32	15
September (Sep)	2018	2	**191**	69	74	48
October (Oct)	2018	1	**36**	22	12	2
November (Nov)	2018	1	**46**	22	23	1
December (Dec)	2018	1	**605**	283	288	34
January (Jan)	2019	3	**1196**	526	526	144
	Total	***35***	***4838***	***1908*** ♂	***1734***	***1196***
					2930 ♀	
