# Supplementary figures and images for: Culex mosquitoes in a French Guiana zoo: insights on species diversity, feeding habits, and parasitic associations
Source: Parasit Vectors. 2026 May 13;19:274. doi: 10.1186/s13071-026-07377-2 (PMC13339560; doi:10.1186/s13071-026-07377-2)

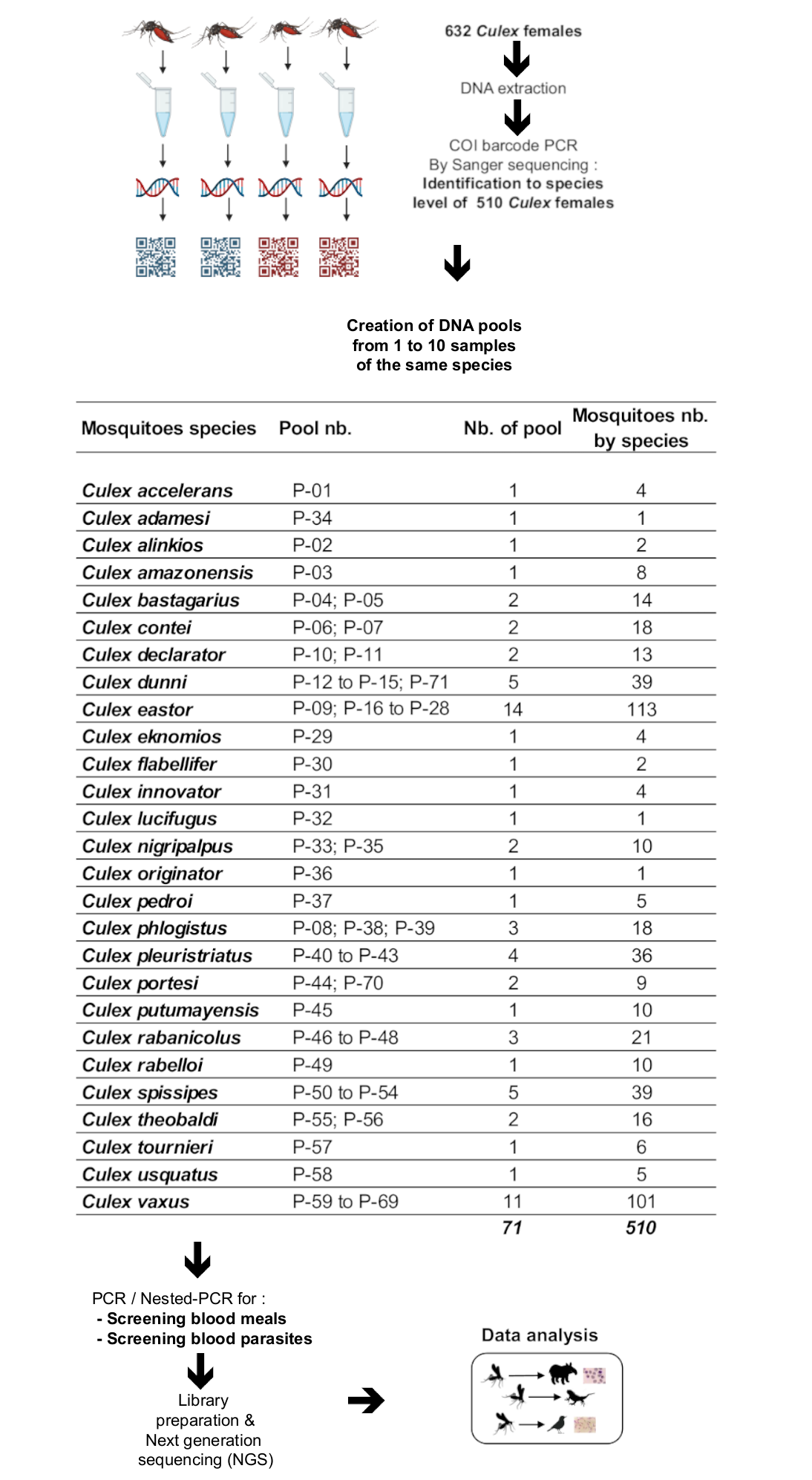

Supplement: Supplementary file 3 — Additional file 3 (PNG 548 KB) [file 13071_2026_7377_MOESM3_ESM.png]
