# Supplementary material for: Culex mosquitoes in a French Guiana zoo: insights on species diversity, feeding habits, and parasitic associations
Source: Parasit Vectors. 2026 May 13;19:274. doi: 10.1186/s13071-026-07377-2 (PMC13339560; doi:10.1186/s13071-026-07377-2)
Supplement: Supplementary file 4 — Additional file 4 (DOCX 72 KB) [file 13071_2026_7377_MOESM4_ESM.docx]

| **Mosquitoes species** | **DNA nb.** | **BOLD Acc. Nb.** | **Identity (%)** | **Sample identification** | **Month of collection** | **GPS-lat** | **GPS-lon** | **Sella Score** | **Associated pool nb** |
| --- | --- | --- | --- | --- | --- | --- | --- | --- | --- |
| ***Culex accelerans*** | Cx_Zoo_0476 | FGMOS2714-20 | 100,00 | 20A30FS2 | Jun | 4,950801032 | -52,489751 | 2 | P-01 |
| ***Culex accelerans*** | Cx_Zoo_0777 | FGMOS2714-20 | 99,70 | 23A36FS1 | Jul | 4,95107797 | -52,49196198 | 2 | P-01 |
| ***Culex accelerans*** | Cx_Zoo_1031 | FGMOS2714-20 | 99,70 | 31A77FS1 | Jan | 4,948672866 | -52,49226876 | 2 | P-01 |
| ***Culex accelerans*** | Cx_Zoo_1125 | FGMOS2714-20 | 100,00 | 33A77FS1 | Jan | 4,947610041 | -52,49244997 | 2 | P-01 |
| ***Culex alinkios*** | Cx_Zoo_0413 | FGMOS2194-20 | 99,24 | 17A22FS1 | Jun | 4,951252732 | -52,49152637 | 2 | P-02 |
| ***Culex alinkios*** | Cx_Zoo_0621 | FGMOS2194-20 | 98,17 | 22A66FS7 | Jul | 4,947476601 | -52,49250521 | 2 | P-02 |
| ***Culex amazonensis*** | Cx_Zoo_0021 | FGMOS2756-20 | 99,39 | 01A26FS1 | Fev | 4,947819924 | -52,49239055 | n.d. | P-03 |
| ***Culex amazonensis*** | Cx_Zoo_0022 | FGMOS2756-20 | 99,85 | 02A7FS1 | Fev | 4,947476601 | -52,49250521 | 2 | P-03 |
| ***Culex amazonensis*** | Cx_Zoo_0183 | FGMOS2756-20 | 100,00 | 10A150FS1 | Avr | 4,951208979 | -52,49116302 | 2 | P-03 |
| ***Culex amazonensis*** | Cx_Zoo_0222 | FGMOS2756-20 | 99,69 | 11A39FS2 | Avr | 4,94896003 | -52,49238996 | 2 | P-03 |
| ***Culex amazonensis*** | Cx_Zoo_0224 | FGMOS2756-20 | 99,85 | 11A12FS2 | Avr | 4,949439978 | -52,49234 | 2 | P-03 |
| ***Culex amazonensis*** | Cx_Zoo_1034 | FGMOS2761-20 | 99,54 | 31A77FS4 | Jan | 4,948672866 | -52,49226876 | 3 | P-03 |
| ***Culex amazonensis*** | Cx_Zoo_1090 | FGMOS2756-20 | 98,94 | 31A29FS1 | Jan | 4,947710037 | -52,49251996 | 2 | P-03 |
| ***Culex amazonensis*** | Cx_Zoo_1176 | FGMOS2756-20 | 99,70 | T1F5FS9 | Jun | 4,950011959 | -52,49289899 | 2 | P-03 |
| ***Culex bastagarius*** | Cx_Zoo_0096 | FGMOS3299-23 | 95,28 | 04A18FS5 | Mar | 4,95107797 | -52,49196198 | 4 | P-04 |
| ***Culex bastagarius*** | Cx_Zoo_0179 | FGMOS3299-23 | 94,22 | 10A11FS3 | Avr | 4,948672866 | -52,49226876 | 2 | P-04 |
| ***Culex bastagarius*** | Cx_Zoo_0264 | FGMOS3299-23 | 95,13 | 11A18FS3 | Avr | 4,947710037 | -52,49251996 | 2 | P-04 |
| ***Culex bastagarius*** | Cx_Zoo_0330 | FGMOS3299-23 | 94,37 | 14A49FS2 | May | 4,949439978 | -52,49234 | 2 | P-04 |
| ***Culex bastagarius*** | Cx_Zoo_0331 | FGMOS3299-23 | 94,37 | 14A49FS3 | May | 4,949439978 | -52,49234 | 4 | P-04 |
| ***Culex bastagarius*** | Cx_Zoo_0360 | FGMOS3299-23 | 95,28 | 15A46FS1 | May | 4,948672866 | -52,49226876 | 2 | P-04 |
| ***Culex bastagarius*** | Cx_Zoo_0464 | FGMOS3299-23 | 95,28 | 20A88FS4 | Jun | 4,947476601 | -52,49250521 | 2 | P-04 |
| ***Culex bastagarius*** | Cx_Zoo_0474 | FGMOS3299-23 | 95,13 | 20A14FS3 | Jun | 4,948672866 | -52,49226876 | 6 | P-04 |
| ***Culex bastagarius*** | Cx_Zoo_1092 | FGMOS3299-23 | 95,28 | 31A29FS3 | Jan | 4,947710037 | -52,49251996 | 2 | P-04 |
| ***Culex bastagarius*** | Cx_Zoo_1184 | FGMOS3299-23 | 94,83 | T1F4FS4 | Jun | 4,950011959 | -52,49289899 | 2 | P-04 |
| ***Culex bastagarius*** | Cx_Zoo_0134 | FGMOS3299-23 | 95,28 | 07A63FS4 | Mar | 4,951839969 | -52,49024997 | 6 | P-05 |
| ***Culex bastagarius*** | Cx_Zoo_0562 | FGMOS3299-23 | 95,28 | 21A50FS1 | Jul | 4,951910041 | -52,49021997 | 2 | P-05 |
| ***Culex bastagarius*** | Cx_Zoo_0882 | FGMOS3299-23 | 94,83 | 24A50FS6 | Jul | 4,952010037 | -52,49096998 | 2 | P-05 |
| ***Culex bastagarius*** | Cx_Zoo_0986 | FGMOS3299-23 | 94,37 | 27A06FS22 | Sep | 4,952010037 | -52,49096998 | 6 | P-05 |
| ***Culex contei*** | Cx_Zoo_0104 | FGMOS2742-20 | 99,24 | 05A19FS1 | Mar | 4,947480792 | -52,49245919 | 4 | P-06 |
| ***Culex contei*** | Cx_Zoo_0125 | FGMOS2742-20 | 99,24 | 07A44FS3 | Mar | 4,947610041 | -52,49244997 | 2 | P-06 |
| ***Culex contei*** | Cx_Zoo_0392 | FGMOS2767-20 | 98,94 | 17A19FS1 | Jun | 4,947476601 | -52,49250521 | 2 | P-06 |
| ***Culex contei*** | Cx_Zoo_0554 | FGMOS2742-20 | 99,24 | 21A24FS2 | Jul | 4,947476601 | -52,49250521 | 2 | P-06 |
| ***Culex contei*** | Cx_Zoo_0764 | FGMOS2742-20 | 99,24 | 23A106FS1 | Jul | 4,947480792 | -52,49245919 | 2 | P-06 |
| ***Culex contei*** | Cx_Zoo_0999 | FGMOS2742-20 | 99,24 | 30A44FS1 | Dec | 4,948672866 | -52,49226876 | 2 | P-06 |
| ***Culex contei*** | Cx_Zoo_1030 | FGMOS2767-20 | 99,24 | 31A04FS1 | Jan | 4,947720012 | -52,49257 | 2 | P-06 |
| ***Culex contei*** | Cx_Zoo_1128 | FGMOS2767-20 | 99,24 | 33A77FS4 | Jan | 4,947610041 | -52,49244997 | 4 | P-06 |
| ***Culex contei*** | Cx_Zoo_1200 | FGMOS2767-20 | 98,63 | 12_RS1_1 | Jun | 4,947819924 | -52,49239055 | 2 | P-06 |
| ***Culex contei*** | Cx_Zoo_0181 | FGMOS2767-20 | 99,24 | 10A08FS1 | Avr | 4,94896003 | -52,49238996 | 4 | P-07 |
| ***Culex contei*** | Cx_Zoo_0228 | FGMOS2767-20 | 98,94 | 11A12FS6 | Avr | 4,949439978 | -52,49234 | 5 | P-07 |
| ***Culex contei*** | Cx_Zoo_0362 | FGMOS2767-20 | 98,94 | 15A34FS2 | May | 4,949439978 | -52,49234 | 3 | P-07 |
| ***Culex contei*** | Cx_Zoo_0478 | FGMOS2742-20 | 99,54 | 20A70FS2 | Jun | 4,95107797 | -52,49196198 | 4 | P-07 |
| ***Culex contei*** | Cx_Zoo_0479 | FGMOS2742-20 | 99,70 | 20A82FS1 | Jun | 4,951195987 | -52,49194697 | 3 | P-07 |
| ***Culex contei*** | Cx_Zoo_0928 | FGMOS2767-20 | 99,24 | 25A54FS1 | Aug | 4,948672866 | -52,49226876 | 2 | P-07 |
| ***Culex contei*** | Cx_Zoo_1133 | FGMOS3175-23 | 95,90 | 33A57FS9 | Jan | 4,95107797 | -52,49196198 | 2 | P-07 |
| ***Culex contei*** | Cx_Zoo_1136 | FGMOS3175-23 | 99,09 | 33A57FS12 | Jan | 4,95107797 | -52,49196198 | 2 | P-07 |
| ***Culex contei*** | Cx_Zoo_1178 | FGMOS2742-20 | 99,24 | T1F2FS1 | Jun | 4,950011959 | -52,49289899 | 2 | P-07 |
| ***Culex phlogistus*** | Cx_Zoo_0073 | FGMOS3175-23 | 98,63 | 03A03FS2 | Mar | 4,950780915 | -52,49287686 | n.d. | P-08 |
| ***Culex phlogistus*** | Cx_Zoo_0234 | FGMOS3175-23 | 98,94 | 11A66FS6 | Avr | 4,951252732 | -52,49152637 | 3 | P-08 |
| ***Culex phlogistus*** | Cx_Zoo_0281 | FGMOS3175-23 | 97,41 | 12A26FS3 | Avr | 4,951252732 | -52,49152637 | 2 | P-08 |
| ***Culex phlogistus*** | Cx_Zoo_0365 | FGMOS3175-23 | 99,09 | 15A35FS1 | May | 4,951154999 | -52,49074099 | 2 | P-08 |
| ***Culex phlogistus*** | Cx_Zoo_0379 | FGMOS3175-23 | 98,48 | 16A28FS1 | May | 4,950990966 | -52,49061802 | 2 | P-08 |
| ***Culex phlogistus*** | Cx_Zoo_1105 | FGMOS3175-23 | 94,38 | 32A05FS2 | Jan | 4,951252732 | -52,49152637 | 2 | P-08 |
| ***Culex phlogistus*** | Cx_Zoo_1118 | FGMOS3175-23 | 98,78 | 32A107FS1 | Jan | 4,951505028 | -52,49295498 | 3 | P-08 |
| ***Culex phlogistus*** | Cx_Zoo_1168 | FGMOS3175-23 | 98,78 | 33A74FS13 | Jan | 4,952010037 | -52,49096998 | 6 | P-08 |
| ***Culex eastor*** | Cx_Zoo_0280 | FGMOS2695-20 | 99,54 | 12A26FS2 | Avr | 4,951252732 | -52,49152637 | 2 | P-09 |
| ***Culex declarator*** | Cx_Zoo_0020 | FGMOS2281-20 | 99,54 | 01A29FS1 | Fev | 4,947476601 | -52,49250521 | 2 | P-10 |
| ***Culex declarator*** | Cx_Zoo_0023 | FGMOS2281-20 | 99,39 | 02A7FS2 | Fev | 4,947476601 | -52,49250521 | 2 | P-10 |
| ***Culex declarator*** | Cx_Zoo_0029 | FGMOS2281-20 | 99,39 | 02A03FS2 | Fev | 4,95107797 | -52,49196198 | 4 | P-10 |
| ***Culex declarator*** | Cx_Zoo_0051 | FGMOS2281-20 | 99,39 | 02A04FS1 | Fev | 4,951195987 | -52,49194697 | 2 | P-10 |
| ***Culex declarator*** | Cx_Zoo_0062 | FGMOS2281-20 | 99,54 | 03A16FS1 | Mar | 4,947710037 | -52,49251996 | 6 | P-10 |
| ***Culex declarator*** | Cx_Zoo_0064 | FGMOS2281-20 | 99,39 | 03A02FS1 | Mar | 4,947710037 | -52,49251996 | 2 | P-10 |
| ***Culex declarator*** | Cx_Zoo_0158 | FGMOS2281-20 | 99,24 | 09A30FS1 | Avr | 4,947710037 | -52,49251996 | 3 | P-10 |
| ***Culex declarator*** | Cx_Zoo_0271 | FGMOS2281-20 | 99,09 | 12A36FS1 | Avr | 4,948672866 | -52,49226876 | 2 | P-10 |
| ***Culex declarator*** | Cx_Zoo_0012 | FGMOS2281-20 | 99,39 | 01A18FS5 | Fev | 4,952010037 | -52,49096998 | 2 | P-11 |
| ***Culex declarator*** | Cx_Zoo_0013 | FGMOS2281-20 | 99,24 | 01A18FS6 | Fev | 4,952010037 | -52,49096998 | 2 | P-11 |
| ***Culex declarator*** | Cx_Zoo_0014 | FGMOS2281-20 | 99,39 | 01A19FS1 | Fev | 4,950780915 | -52,49287686 | 3 | P-11 |
| ***Culex declarator*** | Cx_Zoo_0015 | FGMOS2281-20 | 99,39 | 01A19FS2 | Fev | 4,950780915 | -52,49287686 | 2 | P-11 |
| ***Culex declarator*** | Cx_Zoo_0049 | FGMOS2281-20 | 99,24 | 02A02FS7 | Fev | 4,952010037 | -52,49096998 | 2 | P-11 |
| ***Culex dunni*** | Cx_Zoo_0025 | FGMOS2748-20 | 95,15 | 02A7FS4 | Fev | 4,947476601 | -52,49250521 | 2 | P-12 |
| ***Culex dunni*** | Cx_Zoo_0052 | FGMOS2748-20 | 99,70 | 02A21FS2 | Fev | 4,947956968 | -52,49246598 | 3 | P-12 |
| ***Culex dunni*** | Cx_Zoo_0063 | FGMOS2750-20 | 98,33 | 03A16FS2 | Mar | 4,947710037 | -52,49251996 | 2 | P-12 |
| ***Culex dunni*** | Cx_Zoo_0065 | FGMOS2750-20 | 99,24 | 03A02FS2 | Mar | 4,947710037 | -52,49251996 | 3 | P-12 |
| ***Culex dunni*** | Cx_Zoo_0066 | FGMOS2748-20 | 99,54 | 03A23FS1 | Mar | 4,948141035 | -52,49244704 | 2 | P-12 |
| ***Culex dunni*** | Cx_Zoo_0067 | FGMOS2750-20 | 99,39 | 03A23FS2 | Mar | 4,948141035 | -52,49244704 | 6 | P-12 |
| ***Culex dunni*** | Cx_Zoo_0068 | FGMOS2748-20 | 99,39 | 03A23FS3 | Mar | 4,948141035 | -52,49244704 | 4 | P-12 |
| ***Culex dunni*** | Cx_Zoo_0069 | FGMOS2750-20 | 98,63 | 03A01FS1 | Mar | 4,947956968 | -52,49246598 | 5 | P-12 |
| ***Culex dunni*** | Cx_Zoo_0988 | FGMOS2748-20 | 99,70 | 27A09bisFS1 | Sep | 4,947889997 | -52,49250001 | 2 | P-12 |
| ***Culex dunni*** | Cx_Zoo_0092 | FGMOS2748-20 | 99,24 | 04A18FS1 | Mar | 4,95107797 | -52,49196198 | 3 | P-13 |
| ***Culex dunni*** | Cx_Zoo_0093 | FGMOS2748-20 | 99,54 | 04A18FS2 | Mar | 4,95107797 | -52,49196198 | 3 | P-13 |
| ***Culex dunni*** | Cx_Zoo_0094 | FGMOS2748-20 | 99,54 | 04A18FS3 | Mar | 4,95107797 | -52,49196198 | 4 | P-13 |
| ***Culex dunni*** | Cx_Zoo_0102 | FGMOS2751-20 | 99,24 | 04A18FS11 | Mar | 4,95107797 | -52,49196198 | 6 | P-13 |
| ***Culex dunni*** | Cx_Zoo_1102 | FGMOS2748-20 | 99,24 | 32A43FS1 | Jan | 4,95107797 | -52,49196198 | 4 | P-13 |
| ***Culex dunni*** | Cx_Zoo_1103 | FGMOS2748-20 | 99,54 | 32A43FS2 | Jan | 4,95107797 | -52,49196198 | 2 | P-13 |
| ***Culex dunni*** | Cx_Zoo_1132 | FGMOS2748-20 | 99,39 | 33A57FS8 | Jan | 4,95107797 | -52,49196198 | 2 | P-13 |
| ***Culex dunni*** | Cx_Zoo_1134 | FGMOS2750-20 | 98,48 | 33A57FS10 | Jan | 4,95107797 | -52,49196198 | 2 | P-13 |
| ***Culex dunni*** | Cx_Zoo_1140 | FGMOS2748-20 | 99,70 | 33A57FS16 | Jan | 4,95107797 | -52,49196198 | 3 | P-13 |
| ***Culex dunni*** | Cx_Zoo_0007 | FGMOS2748-20 | 99,54 | 01A17FS3 | Fev | 4,952540025 | -52,49158002 | n.d. | P-14 |
| ***Culex dunni*** | Cx_Zoo_0008 | FGMOS2748-20 | 99,39 | 01A18FS1 | Fev | 4,952010037 | -52,49096998 | n.d. | P-14 |
| ***Culex dunni*** | Cx_Zoo_0011 | FGMOS2750-20 | 99,54 | 01A18FS4 | Fev | 4,952010037 | -52,49096998 | 2 | P-14 |
| ***Culex dunni*** | Cx_Zoo_0038 | FGMOS2748-20 | 98,43 | 02A24FS2 | Fev | 4,952010037 | -52,49096998 | 2 | P-14 |
| ***Culex dunni*** | Cx_Zoo_0040 | FGMOS2748-20 | 98,63 | 02A12FS2 | Fev | 4,952540025 | -52,49158002 | 2 | P-14 |
| ***Culex dunni*** | Cx_Zoo_0042 | FGMOS2750-20 | 99,85 | 02A12FS7 | Fev | 4,952540025 | -52,49158002 | 2 | P-14 |
| ***Culex dunni*** | Cx_Zoo_0048 | FGMOS2750-20 | 99,39 | 02A02FS6 | Fev | 4,952010037 | -52,49096998 | 2 | P-14 |
| ***Culex dunni*** | Cx_Zoo_0056 | FGMOS2748-20 | 99,70 | 02A10FS3 | Fev | 4,950780915 | -52,49287686 | 2 | P-14 |
| ***Culex dunni*** | Cx_Zoo_0060 | FGMOS2748-20 | 99,39 | 02A10FS7 | Fev | 4,950780915 | -52,49287686 | 2 | P-14 |
| ***Culex dunni*** | Cx_Zoo_0072 | FGMOS2748-20 | 98,33 | 03A03FS1 | Mar | 4,950780915 | -52,49287686 | 6 | P-14 |
| ***Culex dunni*** | Cx_Zoo_0249 | FGMOS2750-20 | 99,70 | 11A53FS3 | Avr | 4,950540019 | -52,49286002 | 2 | P-15 |
| ***Culex dunni*** | Cx_Zoo_0441 | FGMOS2748-20 | 99,54 | 18A60FS3 | Jun | 4,950801032 | -52,489751 | 2 | P-15 |
| ***Culex dunni*** | Cx_Zoo_0443 | FGMOS2748-20 | 99,54 | 18A60FS5 | Jun | 4,950801032 | -52,489751 | 2 | P-15 |
| ***Culex dunni*** | Cx_Zoo_0973 | FGMOS2751-20 | 99,39 | 27A06FS9 | Sep | 4,952010037 | -52,49096998 | 2 | P-15 |
| ***Culex dunni*** | Cx_Zoo_0976 | FGMOS2750-20 | 99,85 | 27A06FS12 | Sep | 4,952010037 | -52,49096998 | 2 | P-15 |
| ***Culex dunni*** | Cx_Zoo_1022 | FGMOS2748-20 | 99,39 | 30A49FS3 | Dec | 4,950540019 | -52,49286002 | 5 | P-15 |
| ***Culex dunni*** | Cx_Zoo_1076 | FGMOS2748-20 | 99,24 | 31A51FS4 | Jan | 4,952010037 | -52,49096998 | 2 | P-15 |
| ***Culex eastor*** | Cx_Zoo_0123 | FGMOS2752-20 | 98,63 | 07A44FS1 | Mar | 4,947610041 | -52,49244997 | 2 | P-16 |
| ***Culex eastor*** | Cx_Zoo_0178 | FGMOS2743-20 | 100,00 | 10A11FS2 | Avr | 4,948672866 | -52,49226876 | 2 | P-16 |
| ***Culex eastor*** | Cx_Zoo_0217 | FGMOS2752-20 | 100,00 | 11A16FS2 | Avr | 4,948672866 | -52,49226876 | 2 | P-16 |
| ***Culex eastor*** | Cx_Zoo_0321 | FGMOS2752-20 | 99,85 | 14A48FS1 | May | 4,947720012 | -52,49257 | 2 | P-16 |
| ***Culex eastor*** | Cx_Zoo_0557 | FGMOS2752-20 | 100,00 | 21A10FS1 | Jul | 4,947720012 | -52,49257 | 4 | P-16 |
| ***Culex eastor*** | Cx_Zoo_0558 | FGMOS2752-20 | 100,00 | 21A108FS2 | Jul | 4,948672866 | -52,49226876 | 2 | P-16 |
| ***Culex eastor*** | Cx_Zoo_0622 | FGMOS2752-20 | 99,85 | 22A66FS8 | Jul | 4,947476601 | -52,49250521 | 2 | P-16 |
| ***Culex eastor*** | Cx_Zoo_0636 | FGMOS2752-20 | 100,00 | 22A34FS1 | Jul | 4,948672866 | -52,49226876 | 2 | P-16 |
| ***Culex eastor*** | Cx_Zoo_0766 | FGMOS2711-20 | 100,00 | 23A107FS2 | Jul | 4,947610041 | -52,49244997 | 2 | P-17 |
| ***Culex eastor*** | Cx_Zoo_0769 | FGMOS2695-20 | 99,54 | 23A24FS1 | Jul | 4,948672866 | -52,49226876 | 2 | P-17 |
| ***Culex eastor*** | Cx_Zoo_0839 | FGMOS2695-20 | 100,00 | 24A42FS1 | Jul | 4,947476601 | -52,49250521 | 2 | P-17 |
| ***Culex eastor*** | Cx_Zoo_0993 | FGMOS2752-20 | 99,85 | 30A64FS2 | Dec | 4,947476601 | -52,49250521 | 2 | P-17 |
| ***Culex eastor*** | Cx_Zoo_1032 | FGMOS2752-20 | 100,00 | 31A77FS2 | Jan | 4,948672866 | -52,49226876 | 2 | P-17 |
| ***Culex eastor*** | Cx_Zoo_1097 | FGMOS2752-20 | 100,00 | 32A91FS1 | Jan | 4,947610041 | -52,49244997 | 2 | P-17 |
| ***Culex eastor*** | Cx_Zoo_1206 | FGMOS2752-20 | 100,00 | 16_RS2_1 | Jun | 4,947819924 | -52,49239055 | 2 | P-17 |
| ***Culex eastor*** | Cx_Zoo_1208 | FGMOS2752-20 | 100,00 | 16_RS2_3 | Jun | 4,947819924 | -52,49239055 | 2 | P-17 |
| ***Culex eastor*** | Cx_Zoo_0105 | FGMOS2752-20 | 100,00 | 05A13FS1 | Mar | 4,951195987 | -52,49194697 | n.d. | P-18 |
| ***Culex eastor*** | Cx_Zoo_0328 | FGMOS2752-20 | 100,00 | 14A28FS1 | May | 4,94896003 | -52,49238996 | 3 | P-18 |
| ***Culex eastor*** | Cx_Zoo_0778 | FGMOS2743-20 | 100,00 | 23A60FS1 | Jul | 4,951195987 | -52,49194697 | 2 | P-18 |
| ***Culex eastor*** | Cx_Zoo_0868 | FGMOS2743-20 | 100,00 | 24A08FS5 | Jul | 4,948672866 | -52,49226876 | 2 | P-18 |
| ***Culex eastor*** | Cx_Zoo_0939 | FGMOS2743-20 | 100,00 | 25A01FS3 | Aug | 4,948890042 | -52,49287996 | 2 | P-18 |
| ***Culex eastor*** | Cx_Zoo_1170 | FGMOS2752-20 | 100,00 | T1F5FS1 | Jun | 4,950011959 | -52,49289899 | 2 | P-18 |
| ***Culex eastor*** | Cx_Zoo_1172 | FGMOS2752-20 | 99,39 | T1F5FS5 | Jun | 4,950011959 | -52,49289899 | 2 | P-18 |
| ***Culex eastor*** | Cx_Zoo_1174 | FGMOS2752-20 | 100,00 | T1F5FS7 | Jun | 4,950011959 | -52,49289899 | 2 | P-18 |
| ***Culex eastor*** | Cx_Zoo_0047 | FGMOS2752-20 | 100,00 | 02A02FS5 | Fev | 4,952010037 | -52,49096998 | 2 | P-19 |
| ***Culex eastor*** | Cx_Zoo_0241 | FGMOS2752-20 | 100,00 | 11A33FS5 | Avr | 4,952010037 | -52,49096998 | 2 | P-19 |
| ***Culex eastor*** | Cx_Zoo_0242 | FGMOS2743-20 | 99,70 | 11A33FS6 | Avr | 4,952010037 | -52,49096998 | 2 | P-19 |
| ***Culex eastor*** | Cx_Zoo_0316 | FGMOS2695-20 | 100,00 | 13A55FS1 | May | 4,951839969 | -52,49024997 | 2 | P-19 |
| ***Culex eastor*** | Cx_Zoo_0382 | FGMOS2752-20 | 99,85 | 16A32FS1 | May | 4,952010037 | -52,49096998 | 2 | P-19 |
| ***Culex eastor*** | Cx_Zoo_0449 | FGMOS2743-20 | 100,00 | 19A38FS1 | Jun | 4,950801032 | -52,489751 | 2 | P-19 |
| ***Culex eastor*** | Cx_Zoo_0477 | FGMOS2752-20 | 100,00 | 20A30FS3 | Jun | 4,950801032 | -52,489751 | 2 | P-19 |
| ***Culex eastor*** | Cx_Zoo_0504 | FGMOS2752-20 | 100,00 | 20A47FS11 | Jun | 4,952010037 | -52,49096998 | 2 | P-19 |
| ***Culex eastor*** | Cx_Zoo_0508 | FGMOS2752-20 | 100,00 | 20A47FS15 | Jun | 4,952010037 | -52,49096998 | 2 | P-19 |
| ***Culex eastor*** | Cx_Zoo_0509 | FGMOS2752-20 | 100,00 | 20A47FS16 | Jun | 4,952010037 | -52,49096998 | 2 | P-19 |
| ***Culex eastor*** | Cx_Zoo_0546 | FGMOS2752-20 | 100,00 | 20A95FS3 | Jun | 4,952540025 | -52,49158002 | 2 | P-20 |
| ***Culex eastor*** | Cx_Zoo_0560 | FGMOS2752-20 | 99,70 | 21A36FS1 | Jul | 4,951252732 | -52,49152637 | 2 | P-20 |
| ***Culex eastor*** | Cx_Zoo_0688 | FGMOS2695-20 | 100,00 | 22A02FS40 | Jul | 4,952010037 | -52,49096998 | 2 | P-20 |
| ***Culex eastor*** | Cx_Zoo_0690 | FGMOS2695-20 | 100,00 | 22A02FS42 | Jul | 4,952010037 | -52,49096998 | 2 | P-20 |
| ***Culex eastor*** | Cx_Zoo_0693 | FGMOS2752-20 | 100,00 | 22A02FS45 | Jul | 4,952010037 | -52,49096998 | 2 | P-20 |
| ***Culex eastor*** | Cx_Zoo_0696 | FGMOS2752-20 | 100,00 | 22A02FS48 | Jul | 4,952010037 | -52,49096998 | 4 | P-20 |
| ***Culex eastor*** | Cx_Zoo_0697 | FGMOS2752-20 | 100,00 | 22A33FS1 | Jul | 4,952010037 | -52,49096998 | 2 | P-20 |
| ***Culex eastor*** | Cx_Zoo_0785 | FGMOS2752-20 | 99,85 | 23A79FS5 | Jul | 4,952010037 | -52,49096998 | 2 | P-20 |
| ***Culex eastor*** | Cx_Zoo_0786 | FGMOS2752-20 | 100,00 | 23A79FS6 | Jul | 4,952010037 | -52,49096998 | 2 | P-20 |
| ***Culex eastor*** | Cx_Zoo_0791 | FGMOS2752-20 | 100,00 | 23A79FS11 | Jul | 4,952010037 | -52,49096998 | 2 | P-20 |
| ***Culex eastor*** | Cx_Zoo_0797 | FGMOS2752-20 | 99,70 | 23A79FS17 | Jul | 4,952010037 | -52,49096998 | 2 | P-21 |
| ***Culex eastor*** | Cx_Zoo_0798 | FGMOS2752-20 | 100,00 | 23A79FS18 | Jul | 4,952010037 | -52,49096998 | 2 | P-21 |
| ***Culex eastor*** | Cx_Zoo_0881 | FGMOS2752-20 | 100,00 | 24A50FS5 | Jul | 4,952010037 | -52,49096998 | 2 | P-21 |
| ***Culex eastor*** | Cx_Zoo_0884 | FGMOS2752-20 | 100,00 | 24A50FS8 | Jul | 4,952010037 | -52,49096998 | 2 | P-21 |
| ***Culex eastor*** | Cx_Zoo_0885 | FGMOS2743-20 | 99,70 | 24A50FS9 | Jul | 4,952010037 | -52,49096998 | 2 | P-21 |
| ***Culex eastor*** | Cx_Zoo_0949 | FGMOS2752-20 | 99,85 | 26A45FS3 | Sep | 4,950801032 | -52,489751 | 2 | P-21 |
| ***Culex eastor*** | Cx_Zoo_1005 | FGMOS2752-20 | 100,00 | 30A74FS1 | Dec | 4,951252732 | -52,49152637 | 2 | P-21 |
| ***Culex eastor*** | Cx_Zoo_1006 | FGMOS2743-20 | 100,00 | 30A92FS1 | Dec | 4,951252732 | -52,49152637 | 2 | P-21 |
| ***Culex eastor*** | Cx_Zoo_1008 | FGMOS2743-20 | 100,00 | 30A59FS1 | Dec | 4,950990966 | -52,49061802 | 2 | P-21 |
| ***Culex eastor*** | Cx_Zoo_1014 | FGMOS2743-20 | 100,00 | 30A59FS7 | Dec | 4,950990966 | -52,49061802 | 2 | P-21 |
| ***Culex eastor*** | Cx_Zoo_1015 | FGMOS2743-20 | 100,00 | 30A102FS1 | Dec | 4,952010037 | -52,49096998 | 6 | P-22 |
| ***Culex eastor*** | Cx_Zoo_1043 | FGMOS2743-20 | 100,00 | 31A49FS3 | Jan | 4,952010037 | -52,49096998 | 4 | P-22 |
| ***Culex eastor*** | Cx_Zoo_1070 | FGMOS2743-20 | 99,54 | 31A22FS30 | Jan | 4,952010037 | -52,49096998 | 5 | P-22 |
| ***Culex eastor*** | Cx_Zoo_1072 | FGMOS2743-20 | 100,00 | 31A22FS32 | Jan | 4,952010037 | -52,49096998 | 5 | P-22 |
| ***Culex eastor*** | Cx_Zoo_1073 | FGMOS2752-20 | 100,00 | 31A51FS1 | Jan | 4,952010037 | -52,49096998 | 2 | P-22 |
| ***Culex eastor*** | Cx_Zoo_1074 | FGMOS2752-20 | 100,00 | 31A51FS2 | Jan | 4,952010037 | -52,49096998 | 2 | P-22 |
| ***Culex eastor*** | Cx_Zoo_1081 | FGMOS2752-20 | 100,00 | 31A51FS9 | Jan | 4,952010037 | -52,49096998 | 2 | P-22 |
| ***Culex eastor*** | Cx_Zoo_1107 | FGMOS2695-20 | 99,85 | 32A82FS1 | Jan | 4,952010037 | -52,49096998 | 2 | P-22 |
| ***Culex eastor*** | Cx_Zoo_0113 | FGMOS2743-20 | 99,70 | 06A56FS1 | Mar | 4,947956968 | -52,49246598 | 2 | P-23 |
| ***Culex eastor*** | Cx_Zoo_0127 | FGMOS2695-20 | 99,85 | 07A44FS5 | Mar | 4,947610041 | -52,49244997 | 5 | P-23 |
| ***Culex eastor*** | Cx_Zoo_0322 | FGMOS2711-20 | 99,39 | 14A56FS1 | May | 4,947880022 | -52,49241997 | 6 | P-23 |
| ***Culex eastor*** | Cx_Zoo_0327 | FGMOS2743-20 | 99,85 | 14A66FS8 | May | 4,948672866 | -52,49226876 | 5 | P-23 |
| ***Culex eastor*** | Cx_Zoo_0623 | FGMOS2743-20 | 100,00 | 22A66FS9 | Jul | 4,947476601 | -52,49250521 | 2 | P-23 |
| ***Culex eastor*** | Cx_Zoo_0625 | FGMOS2743-20 | 100,00 | 22A66FS11 | Jul | 4,947476601 | -52,49250521 | 4 | P-23 |
| ***Culex eastor*** | Cx_Zoo_0626 | FGMOS2743-20 | 100,00 | 22A75FS1 | Jul | 4,947480792 | -52,49245919 | 2 | P-23 |
| ***Culex eastor*** | Cx_Zoo_0838 | FGMOS2695-20 | 99,70 | 23A92FS4 | Jul | 4,947330002 | -52,49211998 | 2 | P-23 |
| ***Culex eastor*** | Cx_Zoo_0844 | FGMOS2711-20 | 99,70 | 24A94FS1 | Jul | 4,947610041 | -52,49244997 | 2 | P-23 |
| ***Culex eastor*** | Cx_Zoo_0845 | FGMOS2695-20 | 99,54 | 24A94FS2 | Jul | 4,947610041 | -52,49244997 | 2 | P-23 |
| ***Culex eastor*** | Cx_Zoo_0920 | FGMOS2695-20 | 99,70 | 24A74FS1 | Jul | 4,947330002 | -52,49211998 | 2 | P-24 |
| ***Culex eastor*** | Cx_Zoo_0943 | FGMOS2695-20 | 99,54 | 26A72FS3 | Sep | 4,947476601 | -52,49250521 | 2 | P-24 |
| ***Culex eastor*** | Cx_Zoo_0996 | FGMOS2695-20 | 99,70 | 30A08FS2 | Dec | 4,947610041 | -52,49244997 | 3 | P-24 |
| ***Culex eastor*** | Cx_Zoo_0997 | FGMOS2743-20 | 99,85 | 30A08FS3 | Dec | 4,947610041 | -52,49244997 | 3 | P-24 |
| ***Culex eastor*** | Cx_Zoo_1089 | FGMOS2695-20 | 99,85 | 31A35FS1 | Jan | 4,947710037 | -52,49251996 | 2 | P-24 |
| ***Culex eastor*** | Cx_Zoo_1099 | FGMOS2743-20 | 99,70 | 32A99FS1 | Jan | 4,948672866 | -52,49226876 | 2 | P-24 |
| ***Culex eastor*** | Cx_Zoo_1127 | FGMOS2695-20 | 99,70 | 33A77FS3 | Jan | 4,947610041 | -52,49244997 | 3 | P-24 |
| ***Culex eastor*** | Cx_Zoo_1192 | FGMOS2695-20 | 99,54 | T2F6FS4 | Jun | 4,947819924 | -52,49239055 | 2 | P-24 |
| ***Culex eastor*** | Cx_Zoo_0408 | FGMOS2743-20 | 100,00 | 17A29FS2 | Jun | 4,949439978 | -52,49234 | 4 | P-25 |
| ***Culex eastor*** | Cx_Zoo_0867 | FGMOS2695-20 | 99,54 | 24A08FS4 | Jul | 4,948672866 | -52,49226876 | 2 | P-25 |
| ***Culex eastor*** | Cx_Zoo_0869 | FGMOS2743-20 | 99,85 | 24A08FS6 | Jul | 4,948672866 | -52,49226876 | 2 | P-25 |
| ***Culex eastor*** | Cx_Zoo_1001 | FGMOS2695-20 | 99,54 | 30A02FS1 | Dec | 4,95107797 | -52,49196198 | 2 | P-25 |
| ***Culex eastor*** | Cx_Zoo_0238 | FGMOS2743-20 | 100,00 | 11A33FS1 | Avr | 4,952010037 | -52,49096998 | 2 | P-26 |
| ***Culex eastor*** | Cx_Zoo_0240 | FGMOS2743-20 | 100,00 | 11A33FS3 | Avr | 4,952010037 | -52,49096998 | 2 | P-26 |
| ***Culex eastor*** | Cx_Zoo_0337 | FGMOS2743-20 | 99,85 | 14A38FS1 | May | 4,951208979 | -52,49116302 | 2 | P-26 |
| ***Culex eastor*** | Cx_Zoo_0450 | FGMOS2695-20 | 99,85 | 19A110FS1 | Jun | 4,952010037 | -52,49096998 | 6 | P-26 |
| ***Culex eastor*** | Cx_Zoo_0505 | FGMOS2743-20 | 100,00 | 20A47FS12 | Jun | 4,952010037 | -52,49096998 | 2 | P-26 |
| ***Culex eastor*** | Cx_Zoo_0561 | FGMOS2743-20 | 99,39 | 21A36FS2 | Jul | 4,951252732 | -52,49152637 | 2 | P-26 |
| ***Culex eastor*** | Cx_Zoo_0687 | FGMOS2743-20 | 100,00 | 22A02FS39 | Jul | 4,952010037 | -52,49096998 | 2 | P-26 |
| ***Culex eastor*** | Cx_Zoo_0689 | FGMOS2743-20 | 100,00 | 22A02FS41 | Jul | 4,952010037 | -52,49096998 | 2 | P-26 |
| ***Culex eastor*** | Cx_Zoo_0692 | FGMOS2711-20 | 99,85 | 22A02FS44 | Jul | 4,952010037 | -52,49096998 | 2 | P-26 |
| ***Culex eastor*** | Cx_Zoo_0694 | FGMOS2743-20 | 100,00 | 22A02FS46 | Jul | 4,952010037 | -52,49096998 | 3 | P-26 |
| ***Culex eastor*** | Cx_Zoo_0795 | FGMOS2743-20 | 100,00 | 23A79FS15 | Jul | 4,952010037 | -52,49096998 | 2 | P-27 |
| ***Culex eastor*** | Cx_Zoo_0796 | FGMOS2743-20 | 100,00 | 23A79FS16 | Jul | 4,952010037 | -52,49096998 | 2 | P-27 |
| ***Culex eastor*** | Cx_Zoo_0876 | FGMOS2743-20 | 99,85 | 24A18FS1 | Jul | 4,951108983 | -52,48995602 | 2 | P-27 |
| ***Culex eastor*** | Cx_Zoo_0877 | FGMOS2695-20 | 99,85 | 24A18FS2 | Jul | 4,951108983 | -52,48995602 | 2 | P-27 |
| ***Culex eastor*** | Cx_Zoo_0880 | FGMOS2743-20 | 99,85 | 24A50FS4 | Jul | 4,952010037 | -52,49096998 | 2 | P-27 |
| ***Culex eastor*** | Cx_Zoo_0883 | FGMOS2695-20 | 99,85 | 24A50FS7 | Jul | 4,952010037 | -52,49096998 | 2 | P-27 |
| ***Culex eastor*** | Cx_Zoo_0934 | FGMOS2743-20 | 99,70 | 25A83FS1 | Aug | 4,950780915 | -52,49287686 | 2 | P-27 |
| ***Culex eastor*** | Cx_Zoo_0936 | FGMOS2743-20 | 99,85 | 25A83FS3 | Aug | 4,950780915 | -52,49287686 | 2 | P-27 |
| ***Culex eastor*** | Cx_Zoo_0977 | FGMOS2743-20 | 100,00 | 27A06FS13 | Sep | 4,952010037 | -52,49096998 | 2 | P-27 |
| ***Culex eastor*** | Cx_Zoo_1009 | FGMOS2743-20 | 99,54 | 30A59FS2 | Dec | 4,950990966 | -52,49061802 | 2 | P-27 |
| ***Culex eastor*** | Cx_Zoo_1011 | FGMOS2743-20 | 99,70 | 30A59FS4 | Dec | 4,950990966 | -52,49061802 | 2 | P-28 |
| ***Culex eastor*** | Cx_Zoo_1040 | FGMOS2743-20 | 99,70 | 31A28FS1 | Jan | 4,951154999 | -52,49074099 | 2 | P-28 |
| ***Culex eastor*** | Cx_Zoo_1071 | FGMOS2743-20 | 100,00 | 31A22FS31 | Jan | 4,952010037 | -52,49096998 | 5 | P-28 |
| ***Culex eastor*** | Cx_Zoo_1077 | FGMOS2743-20 | 100,00 | 31A51FS5 | Jan | 4,952010037 | -52,49096998 | 2 | P-28 |
| ***Culex eastor*** | Cx_Zoo_1079 | FGMOS2743-20 | 99,70 | 31A51FS7 | Jan | 4,952010037 | -52,49096998 | 2 | P-28 |
| ***Culex eastor*** | Cx_Zoo_1080 | FGMOS2743-20 | 100,00 | 31A51FS8 | Jan | 4,952010037 | -52,49096998 | 2 | P-28 |
| ***Culex eastor*** | Cx_Zoo_1117 | FGMOS2743-20 | 99,85 | 32A58FS1 | Jan | 4,952540025 | -52,49158002 | 2 | P-28 |
| ***Culex eastor*** | Cx_Zoo_1164 | FGMOS2743-20 | 99,85 | 33A74FS9 | Jan | 4,952010037 | -52,49096998 | 2 | P-28 |
| ***Culex eknomios*** | Cx_Zoo_0309 | FGMOS2440-20 | 100,00 | 13A27FS2 | May | 4,947610041 | -52,49244997 | 2 | P-29 |
| ***Culex eknomios*** | Cx_Zoo_0358 | FGMOS2440-20 | 99,24 | 14A11FS3 | May | 4,947950011 | -52,49257998 | 4 | P-29 |
| ***Culex eknomios*** | Cx_Zoo_0359 | FGMOS2440-20 | 100,00 | 14A26FS1 | May | 4,947710037 | -52,49251996 | 5 | P-29 |
| ***Culex eknomios*** | Cx_Zoo_0463 | FGMOS2440-20 | 100,00 | 20A88FS3 | Jun | 4,947476601 | -52,49250521 | 2 | P-29 |
| ***Culex flabellifer*** | Cx_Zoo_1106 | FGMOS2225-20 | 93,77 | 32A05FS3 | Jan | 4,951252732 | -52,49152637 | 2 | P-30 |
| ***Culex flabellifer*** | Cx_Zoo_1124 | FGMOS2225-20 | 94,53 | 33A03FS1 | Jan | 4,947480792 | -52,49245919 | 2 | P-30 |
| ***Culex innovator*** | Cx_Zoo_0182 | FGMOS2221-20 | 98,48 | 10A43FS1 | Avr | 4,951195987 | -52,49194697 | 4 | P-31 |
| ***Culex innovator*** | Cx_Zoo_0334 | FGMOS2204-20 | 99,39 | 14A16FS1 | May | 4,951195987 | -52,49194697 | 2 | P-31 |
| ***Culex innovator*** | Cx_Zoo_0341 | FGMOS2221-20 | 99,09 | 14A51FS1 | May | 4,952540025 | -52,49158002 | 6 | P-31 |
| ***Culex innovator*** | Cx_Zoo_0389 | FGMOS2221-20 | 98,78 | 16A33FS4 | May | 4,950540019 | -52,49286002 | 6 | P-31 |
| ***Culex lucifugus*** | Cx_Zoo_0282 | FGMOS2001-20 | 98,18 | 12A26FS4 | Avr | 4,951252732 | -52,49152637 | 3 | P-32 |
| ***Culex nigripalpus*** | Cx_Zoo_0338 | FGMOS2667-20 | 99,09 | 14A23FS1 | May | 4,950659964 | -52,49004001 | 6 | P-33 |
| ***Culex nigripalpus*** | Cx_Zoo_0773 | FGMOS2271-20 | 100,00 | 23A24FS5 | Jul | 4,948672866 | -52,49226876 | 2 | P-33 |
| ***Culex nigripalpus*** | Cx_Zoo_0774 | FGMOS2667-20 | 98,18 | 23A24FS6 | Jul | 4,948672866 | -52,49226876 | 2 | P-33 |
| ***Culex adamesi*** | Cx_Zoo_0333 | FGMOS2220-20 | 96,80 | 14A49FS5 | May | 4,949439978 | -52,49234 | 6 | P-34 |
| ***Culex nigripalpus*** | Cx_Zoo_0325 | FGMOS2667-20 | 99,85 | 14A66FS3 | May | 4,948672866 | -52,49226876 | 2 | P-35 |
| ***Culex nigripalpus*** | Cx_Zoo_0695 | FGMOS2667-20 | 100,00 | 22A02FS47 | Jul | 4,952010037 | -52,49096998 | 3 | P-35 |
| ***Culex nigripalpus*** | Cx_Zoo_0871 | FGMOS2667-20 | 99,85 | 24A08FS8 | Jul | 4,948672866 | -52,49226876 | 3 | P-35 |
| ***Culex nigripalpus*** | Cx_Zoo_1000 | FGMOS2667-20 | 100,00 | 30A10FS1 | Dec | 4,94896003 | -52,49238996 | 2 | P-35 |
| ***Culex nigripalpus*** | Cx_Zoo_1137 | FGMOS2667-20 | 100,00 | 33A57FS13 | Jan | 4,95107797 | -52,49196198 | 3 | P-35 |
| ***Culex nigripalpus*** | Cx_Zoo_1138 | FGMOS2667-20 | 99,85 | 33A57FS14 | Jan | 4,95107797 | -52,49196198 | 3 | P-35 |
| ***Culex nigripalpus*** | Cx_Zoo_1210 | FGMOS2667-20 | 99,85 | 6_RS2_3 | Jun | 4,947299073 | -52,49263898 | 6 | P-35 |
| ***Culex originator*** | Cx_Zoo_0354 | FGMOS811-16 | 99,70 | 14A60FS1 | May | 4,950780915 | -52,49287686 | 3 | P-36 |
| ***Culex pedroi*** | Cx_Zoo_0001 | FGMOS2700-20 | 99,85 | 01A01FS2 | Fev | 4,947476601 | -52,49250521 | 2 | P-37 |
| ***Culex pedroi*** | Cx_Zoo_0002 | FGMOS2758-20 | 99,70 | 01A03FS3 | Fev | 4,947710037 | -52,49251996 | n.d. | P-37 |
| ***Culex pedroi*** | Cx_Zoo_0003 | FGMOS2700-20 | 99,39 | 01A03FS4 | Fev | 4,947710037 | -52,49251996 | n.d. | P-37 |
| ***Culex pedroi*** | Cx_Zoo_0070 | FGMOS2700-20 | 99,85 | 03A14FS1 | Mar | 4,947956968 | -52,49246598 | 4 | P-37 |
| ***Culex pedroi*** | Cx_Zoo_0071 | FGMOS2700-20 | 99,85 | 03A10FS1 | Mar | 4,950011959 | -52,49289899 | 2 | P-37 |
| ***Culex phlogistus*** | Cx_Zoo_0221 | FGMOS3175-23 | 99,24 | 11A39FS1 | Avr | 4,94896003 | -52,49238996 | 2 | P-38 |
| ***Culex phlogistus*** | Cx_Zoo_0317 | FGMOS3175-23 | 98,94 | 13A55FS2 | May | 4,951839969 | -52,49024997 | 2 | P-38 |
| ***Culex phlogistus*** | Cx_Zoo_0467 | FGMOS3175-23 | 98,63 | 20A88FS7 | Jun | 4,947476601 | -52,49250521 | 2 | P-38 |
| ***Culex phlogistus*** | Cx_Zoo_0846 | FGMOS3175-23 | 98,94 | 24A94FS3 | Jul | 4,947610041 | -52,49244997 | 2 | P-38 |
| ***Culex phlogistus*** | Cx_Zoo_0975 | FGMOS3175-23 | 99,39 | 27A06FS11 | Sep | 4,952010037 | -52,49096998 | 2 | P-38 |
| ***Culex phlogistus*** | Cx_Zoo_1167 | FGMOS3175-23 | 98,94 | 33A74FS12 | Jan | 4,952010037 | -52,49096998 | 4 | P-38 |
| ***Culex phlogistus*** | Cx_Zoo_0109 | FGMOS1542-20 | 99,09 | 05A04FS2 | Mar | 4,952010037 | -52,49096998 | 4 | P-39 |
| ***Culex phlogistus*** | Cx_Zoo_0278 | FGMOS1542-20 | 98,94 | 12A33FS2 | Avr | 4,95107797 | -52,49196198 | 4 | P-39 |
| ***Culex phlogistus*** | Cx_Zoo_0874 | FGMOS1542-20 | 99,09 | 24A11FS1 | Jul | 4,951252732 | -52,49152637 | 2 | P-39 |
| ***Culex phlogistus*** | Cx_Zoo_1021 | FGMOS1542-20 | 99,09 | 30A49FS2 | Dec | 4,950540019 | -52,49286002 | 3 | P-39 |
| ***Culex pleuristriatus*** | Cx_Zoo_0024 | FGMOS159-16 | 97,57 | 02A7FS3 | Fev | 4,947476601 | -52,49250521 | 2 | P-40 |
| ***Culex pleuristriatus*** | Cx_Zoo_0129 | FGMOS159-16 | 99,54 | 07A65FS1 | Mar | 4,947710037 | -52,49251996 | 6 | P-40 |
| ***Culex pleuristriatus*** | Cx_Zoo_0220 | FGMOS159-16 | 99,54 | 11A16FS5 | Avr | 4,948672866 | -52,49226876 | 4 | P-40 |
| ***Culex pleuristriatus*** | Cx_Zoo_0252 | FGMOS159-16 | 99,85 | 11A25FS1 | Avr | 4,947710037 | -52,49251996 | 2 | P-40 |
| ***Culex pleuristriatus*** | Cx_Zoo_0254 | FGMOS159-16 | 99,85 | 11A25FS3 | Avr | 4,947710037 | -52,49251996 | 3 | P-40 |
| ***Culex pleuristriatus*** | Cx_Zoo_0255 | FGMOS159-16 | 99,54 | 11A25FS4 | Avr | 4,947710037 | -52,49251996 | 4 | P-40 |
| ***Culex pleuristriatus*** | Cx_Zoo_0257 | FGMOS159-16 | 99,85 | 11A25FS6 | Avr | 4,947710037 | -52,49251996 | 5 | P-40 |
| ***Culex pleuristriatus*** | Cx_Zoo_0258 | FGMOS159-16 | 100,00 | 11A25FS7 | Avr | 4,947710037 | -52,49251996 | 5 | P-40 |
| ***Culex pleuristriatus*** | Cx_Zoo_0259 | FGMOS159-16 | 99,54 | 11A25FS8 | Avr | 4,947710037 | -52,49251996 | 5 | P-40 |
| ***Culex pleuristriatus*** | Cx_Zoo_0260 | FGMOS159-16 | 99,39 | 11A25FS9 | Avr | 4,947710037 | -52,49251996 | 6 | P-40 |
| ***Culex pleuristriatus*** | Cx_Zoo_0261 | FGMOS159-16 | 99,54 | 11A25FS10 | Avr | 4,947710037 | -52,49251996 | 6 | P-41 |
| ***Culex pleuristriatus*** | Cx_Zoo_0301 | FGMOS159-16 | 100,00 | 12A66FS3 | Avr | 4,947710037 | -52,49251996 | 3 | P-41 |
| ***Culex pleuristriatus*** | Cx_Zoo_0302 | FGMOS159-16 | 100,00 | 12A66FS4 | Avr | 4,947710037 | -52,49251996 | 4 | P-41 |
| ***Culex pleuristriatus*** | Cx_Zoo_0303 | FGMOS159-16 | 99,54 | 12A66FS5 | Avr | 4,947710037 | -52,49251996 | 5 | P-41 |
| ***Culex pleuristriatus*** | Cx_Zoo_0304 | FGMOS159-16 | 99,85 | 12A66FS6 | Avr | 4,947710037 | -52,49251996 | 5 | P-41 |
| ***Culex pleuristriatus*** | Cx_Zoo_0305 | FGMOS159-16 | 99,85 | 12A66FS7 | Avr | 4,947710037 | -52,49251996 | 6 | P-41 |
| ***Culex pleuristriatus*** | Cx_Zoo_0306 | FGMOS159-16 | 99,70 | 12A66FS8 | Avr | 4,947710037 | -52,49251996 | 6 | P-41 |
| ***Culex pleuristriatus*** | Cx_Zoo_0226 | FGMOS159-16 | 99,85 | 11A12FS4 | Avr | 4,949439978 | -52,49234 | 3 | P-42 |
| ***Culex pleuristriatus*** | Cx_Zoo_0361 | FGMOS159-16 | 99,85 | 15A20FS1 | May | 4,94896003 | -52,49238996 | 3 | P-42 |
| ***Culex pleuristriatus*** | Cx_Zoo_0371 | FGMOS159-16 | 99,70 | 15A51FS1 | May | 4,947710037 | -52,49251996 | 3 | P-42 |
| ***Culex pleuristriatus*** | Cx_Zoo_0372 | FGMOS159-16 | 99,70 | 15A51FS2 | May | 4,947710037 | -52,49251996 | 5 | P-42 |
| ***Culex pleuristriatus*** | Cx_Zoo_0398 | FGMOS159-16 | 99,85 | 17A32FS4 | Jun | 4,947480792 | -52,49245919 | 3 | P-42 |
| ***Culex pleuristriatus*** | Cx_Zoo_0402 | FGMOS159-16 | 99,85 | 17A62FS1 | Jun | 4,948141035 | -52,49244704 | 2 | P-42 |
| ***Culex pleuristriatus*** | Cx_Zoo_0552 | FGMOS159-16 | 99,70 | 20A62FS5 | Jun | 4,947330002 | -52,49211998 | 6 | P-42 |
| ***Culex pleuristriatus*** | Cx_Zoo_0553 | FGMOS159-16 | 99,54 | 21A24FS1 | Jul | 4,947476601 | -52,49250521 | 4 | P-42 |
| ***Culex pleuristriatus*** | Cx_Zoo_0992 | FGMOS159-16 | 99,85 | 30A64FS1 | Dec | 4,947476601 | -52,49250521 | 2 | P-42 |
| ***Culex pleuristriatus*** | Cx_Zoo_1100 | FGMOS159-16 | 100,00 | 32A33FS2 | Jan | 4,94896003 | -52,49238996 | 4 | P-42 |
| ***Culex pleuristriatus*** | Cx_Zoo_0050 | FGMOS159-16 | 97,57 | 02A27FS1 | Fev | 4,951208979 | -52,49116302 | n.d. | P-43 |
| ***Culex pleuristriatus*** | Cx_Zoo_0054 | FGMOS159-16 | 97,57 | 02A10FS1 | Fev | 4,950780915 | -52,49287686 | 2 | P-43 |
| ***Culex pleuristriatus*** | Cx_Zoo_0058 | FGMOS159-16 | 97,57 | 02A10FS5 | Fev | 4,950780915 | -52,49287686 | 2 | P-43 |
| ***Culex pleuristriatus*** | Cx_Zoo_0059 | FGMOS159-16 | 97,57 | 02A10FS6 | Fev | 4,950780915 | -52,49287686 | 2 | P-43 |
| ***Culex pleuristriatus*** | Cx_Zoo_0135 | FGMOS159-16 | 97,57 | 07A57FS1 | Mar | 4,952010037 | -52,49096998 | 2 | P-43 |
| ***Culex pleuristriatus*** | Cx_Zoo_0136 | FGMOS159-16 | 97,57 | 07A57FS2 | Mar | 4,952010037 | -52,49096998 | 2 | P-43 |
| ***Culex pleuristriatus*** | Cx_Zoo_0948 | FGMOS159-16 | 100,00 | 26A22FS1 | Sep | 4,951252732 | -52,49152637 | 5 | P-43 |
| ***Culex pleuristriatus*** | Cx_Zoo_1041 | FGMOS159-16 | 99,54 | 31A49FS1 | Jan | 4,952010037 | -52,49096998 | 2 | P-43 |
| ***Culex pleuristriatus*** | Cx_Zoo_1044 | FGMOS159-16 | 99,39 | 31A49FS4 | Jan | 4,952010037 | -52,49096998 | 5 | P-43 |
| ***Culex portesi*** | Cx_Zoo_0237 | FGMOS2416-20 | 99,85 | 11A63FS2 | Avr | 4,951839969 | -52,49024997 | 5 | P-44 |
| ***Culex portesi*** | Cx_Zoo_0440 | FGMOS2416-20 | 100,00 | 18A60FS2 | Jun | 4,950801032 | -52,489751 | 2 | P-44 |
| ***Culex portesi*** | Cx_Zoo_0985 | FGMOS2416-20 | 100,00 | 27A06FS21 | Sep | 4,952010037 | -52,49096998 | 4 | P-44 |
| ***Culex portesi*** | Cx_Zoo_0989 | FGMOS2416-20 | 100,00 | 28A80FS1 | Oct | 4,947710037 | -52,49251996 | 5 | P-44 |
| ***Culex portesi*** | Cx_Zoo_1020 | FGMOS2416-20 | 100,00 | 30A49FS1 | Dec | 4,950540019 | -52,49286002 | 2 | P-44 |
| ***Culex portesi*** | Cx_Zoo_1025 | FGMOS2416-20 | 100,00 | 30A41FS1 | Dec | 4,947710037 | -52,49251996 | 6 | P-44 |
| ***Culex portesi*** | Cx_Zoo_1130 | FGMOS2416-20 | 100,00 | 33A57FS6 | Jan | 4,95107797 | -52,49196198 | 2 | P-44 |
| ***Culex putumayensis*** | Cx_Zoo_0074 | FGMOS1065-16 | 99,85 | 03A03FS3 | Mar | 4,950780915 | -52,49287686 | n.d. | P-45 |
| ***Culex putumayensis*** | Cx_Zoo_0297 | FGMOS1065-16 | 100,00 | 12A39FS2 | Avr | 4,950780915 | -52,49287686 | 2 | P-45 |
| ***Culex putumayensis*** | Cx_Zoo_0299 | FGMOS1065-16 | 99,70 | 12A39FS4 | Avr | 4,950780915 | -52,49287686 | 2 | P-45 |
| ***Culex putumayensis*** | Cx_Zoo_0315 | FGMOS1065-16 | 100,00 | 13A59FS2 | May | 4,951195987 | -52,49194697 | 6 | P-45 |
| ***Culex putumayensis*** | Cx_Zoo_0363 | FGMOS1065-16 | 100,00 | 15A61FS1 | May | 4,951252732 | -52,49152637 | 2 | P-45 |
| ***Culex putumayensis*** | Cx_Zoo_0442 | FGMOS1065-16 | 99,85 | 18A60FS4 | Jun | 4,950801032 | -52,489751 | 2 | P-45 |
| ***Culex putumayensis*** | Cx_Zoo_0875 | FGMOS1065-16 | 99,54 | 24A11FS2 | Jul | 4,951252732 | -52,49152637 | 4 | P-45 |
| ***Culex putumayensis*** | Cx_Zoo_0979 | FGMOS1065-16 | 99,85 | 27A06FS15 | Sep | 4,952010037 | -52,49096998 | 2 | P-45 |
| ***Culex putumayensis*** | Cx_Zoo_1002 | FGMOS1065-16 | 99,24 | 30A02FS2 | Dec | 4,95107797 | -52,49196198 | 3 | P-45 |
| ***Culex putumayensis*** | Cx_Zoo_1131 | FGMOS1065-16 | 99,70 | 33A57FS7 | Jan | 4,95107797 | -52,49196198 | 2 | P-45 |
| ***Culex rabanicolus*** | Cx_Zoo_0126 | FGMOS2744-20 | 99,54 | 07A44FS4 | Mar | 4,947610041 | -52,49244997 | 3 | P-46 |
| ***Culex rabanicolus*** | Cx_Zoo_0180 | FGMOS2768-20 | 99,70 | 10A11FS4 | Avr | 4,948672866 | -52,49226876 | 2 | P-46 |
| ***Culex rabanicolus*** | Cx_Zoo_0097 | FGMOS2768-20 | 99,39 | 04A18FS6 | Mar | 4,95107797 | -52,49196198 | 5 | P-47 |
| ***Culex rabanicolus*** | Cx_Zoo_0099 | FGMOS2744-20 | 99,70 | 04A18FS8 | Mar | 4,95107797 | -52,49196198 | 5 | P-47 |
| ***Culex rabanicolus*** | Cx_Zoo_0100 | FGMOS2768-20 | 99,70 | 04A18FS9 | Mar | 4,95107797 | -52,49196198 | 5 | P-47 |
| ***Culex rabanicolus*** | Cx_Zoo_0160 | FGMOS2744-20 | 99,54 | 09A33FS1 | Avr | 4,94896003 | -52,49238996 | 2 | P-47 |
| ***Culex rabanicolus*** | Cx_Zoo_0312 | FGMOS2744-20 | 99,54 | 13A08FS2 | May | 4,948672866 | -52,49226876 | 2 | P-47 |
| ***Culex rabanicolus*** | Cx_Zoo_0313 | FGMOS2768-20 | 99,09 | 13A33FS1 | May | 4,949439978 | -52,49234 | 2 | P-47 |
| ***Culex rabanicolus*** | Cx_Zoo_0403 | FGMOS2744-20 | 99,70 | 17A59FS1 | Jun | 4,948672866 | -52,49226876 | 2 | P-47 |
| ***Culex rabanicolus*** | Cx_Zoo_0864 | FGMOS2744-20 | 99,54 | 24A08FS1 | Jul | 4,948672866 | -52,49226876 | 2 | P-47 |
| ***Culex rabanicolus*** | Cx_Zoo_0865 | FGMOS2768-20 | 99,70 | 24A08FS2 | Jul | 4,948672866 | -52,49226876 | 2 | P-47 |
| ***Culex rabanicolus*** | Cx_Zoo_0045 | FGMOS2744-20 | 98,78 | 02A02FS2 | Fev | 4,952010037 | -52,49096998 | 2 | P-48 |
| ***Culex rabanicolus*** | Cx_Zoo_0046 | FGMOS2744-20 | 99,09 | 02A02FS3 | Fev | 4,952010037 | -52,49096998 | 3 | P-48 |
| ***Culex rabanicolus*** | Cx_Zoo_0108 | FGMOS2234-20 | 99,54 | 05A04FS1 | Mar | 4,952010037 | -52,49096998 | 4 | P-48 |
| ***Culex rabanicolus*** | Cx_Zoo_0161 | FGMOS2744-20 | 99,70 | 09A55FS1 | Avr | 4,952540025 | -52,49158002 | 6 | P-48 |
| ***Culex rabanicolus*** | Cx_Zoo_0251 | FGMOS2234-20 | 99,39 | 11A53FS5 | Avr | 4,950540019 | -52,49286002 | 2 | P-48 |
| ***Culex rabanicolus*** | Cx_Zoo_0369 | FGMOS2234-20 | 99,54 | 15A05FS1 | May | 4,950540019 | -52,49286002 | 2 | P-48 |
| ***Culex rabanicolus*** | Cx_Zoo_0380 | FGMOS2744-20 | 99,54 | 16A05FS2 | May | 4,951154999 | -52,49074099 | 3 | P-48 |
| ***Culex rabanicolus*** | Cx_Zoo_0698 | FGMOS2768-20 | 99,70 | 22A33FS2 | Jul | 4,952010037 | -52,49096998 | 2 | P-48 |
| ***Culex rabanicolus*** | Cx_Zoo_0780 | FGMOS2768-20 | 99,70 | 23A06FS3 | Jul | 4,950801032 | -52,489751 | 2 | P-48 |
| ***Culex rabanicolus*** | Cx_Zoo_1023 | FGMOS2744-20 | 99,70 | 30A20FS1 | Dec | 4,950429965 | -52,49277997 | 2 | P-48 |
| ***Culex rabelloi*** | Cx_Zoo_0314 | FGMOS2230-20 | 98,94 | 13A59FS1 | May | 4,951195987 | -52,49194697 | 5 | P-49 |
| ***Culex rabelloi*** | Cx_Zoo_0335 | FGMOS2230-20 | 99,09 | 14A16FS6 | May | 4,951195987 | -52,49194697 | 6 | P-49 |
| ***Culex rabelloi*** | Cx_Zoo_0381 | FGMOS2881-22 | 99,24 | 16A24FS1 | May | 4,950659964 | -52,49004001 | 2 | P-49 |
| ***Culex rabelloi*** | Cx_Zoo_0412 | FGMOS2881-22 | 99,39 | 17A23FS2 | Jun | 4,951195987 | -52,49194697 | 6 | P-49 |
| ***Culex rabelloi*** | Cx_Zoo_0461 | FGMOS2230-20 | 99,09 | 19A44FS1 | Jun | 4,952540025 | -52,49158002 | 2 | P-49 |
| ***Culex rabelloi*** | Cx_Zoo_0961 | FGMOS2881-22 | 99,39 | 26A82FS1 | Sep | 4,952540025 | -52,49158002 | 2 | P-49 |
| ***Culex rabelloi*** | Cx_Zoo_0963 | FGMOS2230-20 | 99,24 | 27A80FS1 | Sep | 4,951839969 | -52,49024997 | 2 | P-49 |
| ***Culex rabelloi*** | Cx_Zoo_0980 | FGMOS2881-22 | 99,39 | 27A06FS16 | Sep | 4,952010037 | -52,49096998 | 2 | P-49 |
| ***Culex rabelloi*** | Cx_Zoo_1202 | FGMOS2230-20 | 99,09 | 1_RS2_5 | Jun | 4,947819924 | -52,49239055 | 2 | P-49 |
| ***Culex rabelloi*** | Cx_Zoo_1204 | FGMOS2881-22 | 99,39 | 1_RS2_8 | Jun | 4,947819924 | -52,49239055 | 2 | P-49 |
| ***Culex spissipes*** | Cx_Zoo_0212 | FGMOS3379-23 | 98,78 | 11A36FS1 | Avr | 4,947476601 | -52,49250521 | 2 | P-50 |
| ***Culex spissipes*** | Cx_Zoo_0548 | FGMOS2701-20 | 99,24 | 20A13FS1 | Jun | 4,947710037 | -52,49251996 | 3 | P-50 |
| ***Culex spissipes*** | Cx_Zoo_0927 | FGMOS2701-20 | 99,24 | 25A10FS1 | Aug | 4,948672866 | -52,49226876 | 2 | P-50 |
| ***Culex spissipes*** | Cx_Zoo_0946 | FGMOS2701-20 | 98,78 | 26A44FS1 | Sep | 4,947720012 | -52,49257 | 2 | P-50 |
| ***Culex spissipes*** | Cx_Zoo_1095 | FGMOS1522-20 | 99,09 | 32A06FS1 | Jan | 4,947476601 | -52,49250521 | 2 | P-50 |
| ***Culex spissipes*** | Cx_Zoo_1096 | FGMOS2701-20 | 98,48 | 32A06FS2 | Jan | 4,947476601 | -52,49250521 | 2 | P-50 |
| ***Culex spissipes*** | Cx_Zoo_0004 | FGMOS2701-20 | 99,24 | 01A09FS1 | Fev | 4,95107797 | -52,49196198 | n.d. | P-51 |
| ***Culex spissipes*** | Cx_Zoo_0005 | FGMOS3379-23 | 99,24 | 01A09FS3 | Fev | 4,95107797 | -52,49196198 | 3 | P-51 |
| ***Culex spissipes*** | Cx_Zoo_0032 | FGMOS2701-20 | 96,95 | 02A22FS3 | Fev | 4,95107797 | -52,49196198 | 2 | P-51 |
| ***Culex spissipes*** | Cx_Zoo_0480 | FGMOS2701-20 | 98,48 | 20A82FS2 | Jun | 4,951195987 | -52,49194697 | 3 | P-51 |
| ***Culex spissipes*** | Cx_Zoo_0481 | FGMOS2701-20 | 98,63 | 20A82FS3 | Jun | 4,951195987 | -52,49194697 | 6 | P-51 |
| ***Culex spissipes*** | Cx_Zoo_0863 | FGMOS2701-20 | 99,24 | 24A52FS2 | Jul | 4,948672866 | -52,49226876 | 6 | P-51 |
| ***Culex spissipes*** | Cx_Zoo_0873 | FGMOS3379-23 | 98,94 | 24A33FS1 | Jul | 4,951195987 | -52,49194697 | 6 | P-51 |
| ***Culex spissipes*** | Cx_Zoo_1180 | FGMOS2701-20 | 97,57 | T1F8FS3 | Jun | 4,950011959 | -52,49289899 | 2 | P-51 |
| ***Culex spissipes*** | Cx_Zoo_0232 | FGMOS2701-20 | 98,78 | 11A66FS4 | Avr | 4,951252732 | -52,49152637 | 2 | P-52 |
| ***Culex spissipes*** | Cx_Zoo_0236 | FGMOS2701-20 | 97,57 | 11A63FS1 | Avr | 4,951839969 | -52,49024997 | 2 | P-52 |
| ***Culex spissipes*** | Cx_Zoo_0239 | FGMOS2701-20 | 99,39 | 11A33FS2 | Avr | 4,952010037 | -52,49096998 | 2 | P-52 |
| ***Culex spissipes*** | Cx_Zoo_0245 | FGMOS2701-20 | 98,78 | 11A26FS3 | Avr | 4,950780915 | -52,49287686 | 5 | P-52 |
| ***Culex spissipes*** | Cx_Zoo_0246 | FGMOS2701-20 | 99,24 | 11A26FS4 | Avr | 4,950780915 | -52,49287686 | 6 | P-52 |
| ***Culex spissipes*** | Cx_Zoo_0279 | FGMOS2701-20 | 98,94 | 12A26FS1 | Avr | 4,951252732 | -52,49152637 | 2 | P-52 |
| ***Culex spissipes*** | Cx_Zoo_0296 | FGMOS1522-20 | 95,44 | 12A39FS1 | Avr | 4,950780915 | -52,49287686 | 3 | P-52 |
| ***Culex spissipes*** | Cx_Zoo_0298 | FGMOS2701-20 | 99,09 | 12A39FS3 | Avr | 4,950780915 | -52,49287686 | 2 | P-52 |
| ***Culex spissipes*** | Cx_Zoo_0307 | FGMOS2701-20 | 99,09 | 12A13FS1 | Avr | 4,951505028 | -52,49295498 | 3 | P-52 |
| ***Culex spissipes*** | Cx_Zoo_0176 | FGMOS3379-23 | 99,70 | 10A22FS1 | Avr | 4,951252732 | -52,49152637 | 2 | P-53 |
| ***Culex spissipes*** | Cx_Zoo_0177 | FGMOS3379-23 | 98,78 | 10A22FS2 | Avr | 4,951252732 | -52,49152637 | 2 | P-53 |
| ***Culex spissipes*** | Cx_Zoo_0368 | FGMOS2701-20 | 98,63 | 15A44FS2 | May | 4,950780915 | -52,49287686 | 2 | P-53 |
| ***Culex spissipes*** | Cx_Zoo_0383 | FGMOS3379-23 | 98,94 | 16A32FS2 | May | 4,952010037 | -52,49096998 | 4 | P-53 |
| ***Culex spissipes*** | Cx_Zoo_0506 | FGMOS2701-20 | 98,17 | 20A47FS13 | Jun | 4,952010037 | -52,49096998 | 2 | P-53 |
| ***Culex spissipes*** | Cx_Zoo_0507 | FGMOS2701-20 | 97,87 | 20A47FS14 | Jun | 4,952010037 | -52,49096998 | 2 | P-53 |
| ***Culex spissipes*** | Cx_Zoo_0779 | FGMOS1522-20 | 98,78 | 23A02FS1 | Jul | 4,951252732 | -52,49152637 | 2 | P-53 |
| ***Culex spissipes*** | Cx_Zoo_0794 | FGMOS2701-20 | 97,11 | 23A79FS14 | Jul | 4,952010037 | -52,49096998 | 2 | P-53 |
| ***Culex spissipes*** | Cx_Zoo_0835 | FGMOS2701-20 | 98,63 | 23A19FS1 | Jul | 4,950780915 | -52,49287686 | 2 | P-53 |
| ***Culex spissipes*** | Cx_Zoo_0964 | FGMOS2701-20 | 98,94 | 27A80FS2 | Sep | 4,951839969 | -52,49024997 | 3 | P-53 |
| ***Culex spissipes*** | Cx_Zoo_1038 | FGMOS2701-20 | 97,42 | 31A72FS2 | Jan | 4,951252732 | -52,49152637 | 4 | P-54 |
| ***Culex spissipes*** | Cx_Zoo_1045 | FGMOS2701-20 | 98,94 | 31A92FS1 | Jan | 4,951839969 | -52,49024997 | 3 | P-54 |
| ***Culex spissipes*** | Cx_Zoo_1078 | FGMOS2701-20 | 97,72 | 31A51FS6 | Jan | 4,952010037 | -52,49096998 | 2 | P-54 |
| ***Culex spissipes*** | Cx_Zoo_1159 | FGMOS2701-20 | 99,09 | 33ARB5FS1 | Jan | 4,950801032 | -52,489751 | 3 | P-54 |
| ***Culex spissipes*** | Cx_Zoo_1169 | FGMOS2701-20 | 98,78 | 33ARB2FS1 | Jan | 4,950780915 | -52,49287686 | 2 | P-54 |
| ***Culex spissipes*** | Cx_Zoo_1186 | FGMOS2701-20 | 97,57 | T1F4FS6 | Jun | 4,95215999 | -52,49077996 | 2 | P-54 |
| ***Culex theobaldi*** | Cx_Zoo_0082 | FGMOS2223-20 | 99,24 | 03A24FS1 | Mar | 4,947476601 | -52,49250521 | n.d. | P-55 |
| ***Culex theobaldi*** | Cx_Zoo_0083 | FGMOS2223-20 | 98,78 | 03A24FS2 | Mar | 4,947476601 | -52,49250521 | n.d. | P-55 |
| ***Culex theobaldi*** | Cx_Zoo_0084 | FGMOS2160-20 | 99,85 | 03A15FS1 | Mar | 4,947610041 | -52,49244997 | n.d. | P-55 |
| ***Culex theobaldi*** | Cx_Zoo_0090 | FGMOS2160-20 | 100,00 | 04A06FS2 | Mar | 4,947710037 | -52,49251996 | 4 | P-55 |
| ***Culex theobaldi*** | Cx_Zoo_0199 | FGMOS2223-20 | 98,33 | 10A12FS1 | Avr | 4,948672866 | -52,49226876 | 2 | P-55 |
| ***Culex theobaldi*** | Cx_Zoo_0310 | FGMOS1596-20 | 100,00 | 13A30FS1 | May | 4,947720012 | -52,49257 | 3 | P-55 |
| ***Culex theobaldi*** | Cx_Zoo_0447 | FGMOS1596-20 | 99,85 | 18A14FS1 | Jun | 4,947299073 | -52,49263898 | 6 | P-55 |
| ***Culex theobaldi*** | Cx_Zoo_0860 | FGMOS2524-20 | 99,70 | 24A75FS1 | Jul | 4,947880022 | -52,49241997 | 4 | P-55 |
| ***Culex theobaldi*** | Cx_Zoo_1024 | FGMOS2223-20 | 99,70 | 30A101FS1 | Dec | 4,948890042 | -52,49287996 | 2 | P-55 |
| ***Culex theobaldi*** | Cx_Zoo_0075 | FGMOS2524-20 | 99,54 | 03A03FS4 | Mar | 4,950780915 | -52,49287686 | n.d. | P-56 |
| ***Culex theobaldi*** | Cx_Zoo_0247 | FGMOS2160-20 | 99,85 | 11A53FS1 | Avr | 4,950540019 | -52,49286002 | 2 | P-56 |
| ***Culex theobaldi*** | Cx_Zoo_0319 | FGMOS2160-20 | 100,00 | 13A66FS1 | May | 4,950990966 | -52,49061802 | 4 | P-56 |
| ***Culex theobaldi*** | Cx_Zoo_0384 | FGMOS2223-20 | 98,94 | 16A32FS3 | May | 4,952010037 | -52,49096998 | 4 | P-56 |
| ***Culex theobaldi*** | Cx_Zoo_0387 | FGMOS2223-20 | 100,00 | 16A33FS1 | May | 4,950540019 | -52,49286002 | 2 | P-56 |
| ***Culex theobaldi*** | Cx_Zoo_0388 | FGMOS2223-20 | 99,39 | 16A33FS3 | May | 4,950540019 | -52,49286002 | 3 | P-56 |
| ***Culex theobaldi*** | Cx_Zoo_0446 | FGMOS2160-20 | 99,85 | 18A27FS2 | Jun | 4,950540019 | -52,49286002 | 2 | P-56 |
| ***Culex tournieri*** | Cx_Zoo_0686 | FGMOS2227-20 | 97,87 | 22A02FS38 | Jul | 4,952010037 | -52,49096998 | 2 | P-57 |
| ***Culex tournieri*** | Cx_Zoo_0847 | FGMOS2227-20 | 97,87 | 24A94FS4 | Jul | 4,947610041 | -52,49244997 | 2 | P-57 |
| ***Culex tournieri*** | Cx_Zoo_0866 | FGMOS2232-20 | 97,26 | 24A08FS3 | Jul | 4,948672866 | -52,49226876 | 2 | P-57 |
| ***Culex tournieri*** | Cx_Zoo_0879 | FGMOS2232-20 | 97,26 | 24A50FS3 | Jul | 4,952010037 | -52,49096998 | 2 | P-57 |
| ***Culex tournieri*** | Cx_Zoo_0978 | FGMOS2227-20 | 98,02 | 27A06FS14 | Sep | 4,952010037 | -52,49096998 | 2 | P-57 |
| ***Culex tournieri*** | Cx_Zoo_0981 | FGMOS2227-20 | 98,02 | 27A06FS17 | Sep | 4,952010037 | -52,49096998 | 3 | P-57 |
| ***Culex usquatus*** | Cx_Zoo_0017 | FGMOS046-16 | 99,85 | 01A19FS4 | Fev | 4,950780915 | -52,49287686 | 2 | P-58 |
| ***Culex usquatus*** | Cx_Zoo_0018 | FGMOS046-16 | 100,00 | 01A19FS5 | Fev | 4,950780915 | -52,49287686 | 3 | P-58 |
| ***Culex usquatus*** | Cx_Zoo_0057 | FGMOS046-16 | 100,00 | 02A10FS4 | Fev | 4,950780915 | -52,49287686 | 3 | P-58 |
| ***Culex usquatus*** | Cx_Zoo_0130 | FGMOS046-16 | 100,00 | 07A23FS1 | Mar | 4,947956968 | -52,49246598 | 2 | P-58 |
| ***Culex usquatus*** | Cx_Zoo_0947 | FGMOS046-16 | 99,85 | 26A48FS1 | Sep | 4,948672866 | -52,49226876 | 2 | P-58 |
| ***Culex vaxus*** | Cx_Zoo_0078 | FGMOS1713-20 | 100,00 | 03A12FS1 | Mar | 4,947476601 | -52,49250521 | 2 | P-59 |
| ***Culex vaxus*** | Cx_Zoo_0079 | FGMOS1713-20 | 100,00 | 03A12FS2 | Mar | 4,947476601 | -52,49250521 | 4 | P-59 |
| ***Culex vaxus*** | Cx_Zoo_0081 | FGMOS1713-20 | 100,00 | 03A13FS2 | Mar | 4,947476601 | -52,49250521 | 5 | P-59 |
| ***Culex vaxus*** | Cx_Zoo_0085 | FGMOS1713-20 | 99,85 | 03A15FS2 | Mar | 4,947610041 | -52,49244997 | n.d. | P-59 |
| ***Culex vaxus*** | Cx_Zoo_0089 | FGMOS1713-20 | 99,85 | 04A06FS1 | Mar | 4,947710037 | -52,49251996 | 3 | P-59 |
| ***Culex vaxus*** | Cx_Zoo_0091 | FGMOS1713-20 | 100,00 | 04A06FS3 | Mar | 4,947710037 | -52,49251996 | 4 | P-59 |
| ***Culex vaxus*** | Cx_Zoo_0124 | FGMOS1713-20 | 98,17 | 07A44FS2 | Mar | 4,947610041 | -52,49244997 | 2 | P-59 |
| ***Culex vaxus*** | Cx_Zoo_0202 | FGMOS1713-20 | 100,00 | 11A58FS2 | Avr | 4,947476601 | -52,49250521 | 2 | P-59 |
| ***Culex vaxus*** | Cx_Zoo_0206 | FGMOS1713-20 | 99,85 | 11A08FS4 | Avr | 4,947610041 | -52,49244997 | 2 | P-59 |
| ***Culex vaxus*** | Cx_Zoo_0209 | FGMOS1713-20 | 99,85 | 11A13FS3 | Avr | 4,947476601 | -52,49250521 | 2 | P-59 |
| ***Culex vaxus*** | Cx_Zoo_0210 | FGMOS1713-20 | 100,00 | 11A13FS4 | Avr | 4,947476601 | -52,49250521 | 2 | P-60 |
| ***Culex vaxus*** | Cx_Zoo_0211 | FGMOS1713-20 | 99,09 | 11A13FS5 | Avr | 4,947476601 | -52,49250521 | 4 | P-60 |
| ***Culex vaxus*** | Cx_Zoo_0214 | FGMOS1713-20 | 100,00 | 11A10FS1 | Avr | 4,947720012 | -52,49257 | 2 | P-60 |
| ***Culex vaxus*** | Cx_Zoo_0216 | FGMOS1713-20 | 100,00 | 11A16FS1 | Avr | 4,948672866 | -52,49226876 | 2 | P-60 |
| ***Culex vaxus*** | Cx_Zoo_0218 | FGMOS1713-20 | 100,00 | 11A16FS3 | Avr | 4,948672866 | -52,49226876 | 2 | P-60 |
| ***Culex vaxus*** | Cx_Zoo_0219 | FGMOS1713-20 | 100,00 | 11A16FS4 | Avr | 4,948672866 | -52,49226876 | 3 | P-60 |
| ***Culex vaxus*** | Cx_Zoo_0256 | FGMOS1713-20 | 99,69 | 11A25FS5 | Avr | 4,947710037 | -52,49251996 | 4 | P-60 |
| ***Culex vaxus*** | Cx_Zoo_0265 | FGMOS1713-20 | 98,78 | 12A59FS2 | Avr | 4,947476601 | -52,49250521 | 2 | P-60 |
| ***Culex vaxus*** | Cx_Zoo_0266 | FGMOS1713-20 | 100,00 | 12A59FS3 | Avr | 4,947476601 | -52,49250521 | 2 | P-60 |
| ***Culex vaxus*** | Cx_Zoo_0268 | FGMOS1713-20 | 100,00 | 12A59FS5 | Avr | 4,947476601 | -52,49250521 | 2 | P-60 |
| ***Culex vaxus*** | Cx_Zoo_0269 | FGMOS1713-20 | 99,85 | 12A35FS1 | Avr | 4,947610041 | -52,49244997 | 2 | P-61 |
| ***Culex vaxus*** | Cx_Zoo_0270 | FGMOS1713-20 | 100,00 | 12A35FS2 | Avr | 4,947610041 | -52,49244997 | 2 | P-61 |
| ***Culex vaxus*** | Cx_Zoo_0272 | FGMOS1713-20 | 99,70 | 12A36FS2 | Avr | 4,948672866 | -52,49226876 | 3 | P-61 |
| ***Culex vaxus*** | Cx_Zoo_0308 | FGMOS1713-20 | 99,85 | 13A27FS1 | May | 4,947610041 | -52,49244997 | 2 | P-61 |
| ***Culex vaxus*** | Cx_Zoo_0320 | FGMOS1713-20 | 99,85 | 14A46FS1 | May | 4,947476601 | -52,49250521 | 2 | P-61 |
| ***Culex vaxus*** | Cx_Zoo_0323 | FGMOS1713-20 | 100,00 | 14A66FS1 | May | 4,948672866 | -52,49226876 | 2 | P-61 |
| ***Culex vaxus*** | Cx_Zoo_0324 | FGMOS1713-20 | 100,00 | 14A66FS2 | May | 4,948672866 | -52,49226876 | 2 | P-61 |
| ***Culex vaxus*** | Cx_Zoo_0326 | FGMOS1713-20 | 99,85 | 14A66FS5 | May | 4,948672866 | -52,49226876 | 3 | P-61 |
| ***Culex vaxus*** | Cx_Zoo_0390 | FGMOS1713-20 | 100,00 | 16A50FS1 | May | 4,947710037 | -52,49251996 | 2 | P-61 |
| ***Culex vaxus*** | Cx_Zoo_0393 | FGMOS1713-20 | 100,00 | 17A19FS2 | Jun | 4,947476601 | -52,49250521 | 6 | P-61 |
| ***Culex vaxus*** | Cx_Zoo_0394 | FGMOS1713-20 | 100,00 | 17A19FS3 | Jun | 4,947476601 | -52,49250521 | 6 | P-62 |
| ***Culex vaxus*** | Cx_Zoo_0395 | FGMOS1713-20 | 99,54 | 17A32FS1 | Jun | 4,947480792 | -52,49245919 | 2 | P-62 |
| ***Culex vaxus*** | Cx_Zoo_0396 | FGMOS1713-20 | 100,00 | 17A32FS2 | Jun | 4,947480792 | -52,49245919 | 2 | P-62 |
| ***Culex vaxus*** | Cx_Zoo_0397 | FGMOS1713-20 | 99,85 | 17A32FS3 | Jun | 4,947480792 | -52,49245919 | 2 | P-62 |
| ***Culex vaxus*** | Cx_Zoo_0399 | FGMOS1713-20 | 99,70 | 17A03FS1 | Jun | 4,947720012 | -52,49257 | 2 | P-62 |
| ***Culex vaxus*** | Cx_Zoo_0401 | FGMOS1713-20 | 100,00 | 17A14FS2 | Jun | 4,948672866 | -52,49226876 | 2 | P-62 |
| ***Culex vaxus*** | Cx_Zoo_0439 | FGMOS1713-20 | 100,00 | 17A26FS1 | Jun | 4,947330002 | -52,49211998 | 2 | P-62 |
| ***Culex vaxus*** | Cx_Zoo_0462 | FGMOS1713-20 | 99,85 | 20A88FS2 | Jun | 4,947476601 | -52,49250521 | 2 | P-62 |
| ***Culex vaxus*** | Cx_Zoo_0468 | FGMOS1713-20 | 100,00 | 20A88FS8 | Jun | 4,947476601 | -52,49250521 | 2 | P-62 |
| ***Culex vaxus*** | Cx_Zoo_0469 | FGMOS1713-20 | 99,85 | 20A88FS9 | Jun | 4,947476601 | -52,49250521 | 2 | P-62 |
| ***Culex vaxus*** | Cx_Zoo_0470 | FGMOS1713-20 | 100,00 | 20A88FS10 | Jun | 4,947476601 | -52,49250521 | 2 | P-63 |
| ***Culex vaxus*** | Cx_Zoo_0471 | FGMOS1713-20 | 100,00 | 20A88FS11 | Jun | 4,947476601 | -52,49250521 | 2 | P-63 |
| ***Culex vaxus*** | Cx_Zoo_0473 | FGMOS1713-20 | 99,85 | 20A49FS1 | Jun | 4,947480792 | -52,49245919 | 2 | P-63 |
| ***Culex vaxus*** | Cx_Zoo_0549 | FGMOS1713-20 | 100,00 | 20A62FS2 | Jun | 4,947330002 | -52,49211998 | 2 | P-63 |
| ***Culex vaxus*** | Cx_Zoo_0551 | FGMOS1713-20 | 99,70 | 20A62FS4 | Jun | 4,947330002 | -52,49211998 | 2 | P-63 |
| ***Culex vaxus*** | Cx_Zoo_0555 | FGMOS1713-20 | 100,00 | 21A24FS3 | Jul | 4,947476601 | -52,49250521 | 4 | P-63 |
| ***Culex vaxus*** | Cx_Zoo_0556 | FGMOS1713-20 | 100,00 | 21A41FS1 | Jul | 4,947480792 | -52,49245919 | 4 | P-63 |
| ***Culex vaxus*** | Cx_Zoo_0755 | FGMOS1713-20 | 100,00 | 22A86FS1 | Jul | 4,947330002 | -52,49211998 | 4 | P-63 |
| ***Culex vaxus*** | Cx_Zoo_0757 | FGMOS1713-20 | 100,00 | 23A27FS2 | Jul | 4,947476601 | -52,49250521 | 2 | P-63 |
| ***Culex vaxus*** | Cx_Zoo_0758 | FGMOS1713-20 | 100,00 | 23A27FS3 | Jul | 4,947476601 | -52,49250521 | 2 | P-63 |
| ***Culex vaxus*** | Cx_Zoo_0759 | FGMOS1713-20 | 100,00 | 23A27FS4 | Jul | 4,947476601 | -52,49250521 | 2 | P-64 |
| ***Culex vaxus*** | Cx_Zoo_0765 | FGMOS1713-20 | 100,00 | 23A107FS1 | Jul | 4,947610041 | -52,49244997 | 2 | P-64 |
| ***Culex vaxus*** | Cx_Zoo_0767 | FGMOS1713-20 | 100,00 | 23A107FS3 | Jul | 4,947610041 | -52,49244997 | 4 | P-64 |
| ***Culex vaxus*** | Cx_Zoo_0771 | FGMOS1713-20 | 99,85 | 23A24FS3 | Jul | 4,948672866 | -52,49226876 | 2 | P-64 |
| ***Culex vaxus*** | Cx_Zoo_0775 | FGMOS1713-20 | 99,54 | 23A24FS7 | Jul | 4,948672866 | -52,49226876 | 4 | P-64 |
| ***Culex vaxus*** | Cx_Zoo_0836 | FGMOS1713-20 | 100,00 | 23A92FS2 | Jul | 4,947330002 | -52,49211998 | 2 | P-64 |
| ***Culex vaxus*** | Cx_Zoo_0837 | FGMOS1713-20 | 100,00 | 23A92FS3 | Jul | 4,947330002 | -52,49211998 | 5 | P-64 |
| ***Culex vaxus*** | Cx_Zoo_0842 | FGMOS1713-20 | 100,00 | 24A42FS4 | Jul | 4,947476601 | -52,49250521 | 3 | P-64 |
| ***Culex vaxus*** | Cx_Zoo_0843 | FGMOS1713-20 | 100,00 | 24A02FS1 | Jul | 4,947480792 | -52,49245919 | 4 | P-64 |
| ***Culex vaxus*** | Cx_Zoo_0848 | FGMOS1713-20 | 100,00 | 24A94FS5 | Jul | 4,947610041 | -52,49244997 | 2 | P-64 |
| ***Culex vaxus*** | Cx_Zoo_0921 | FGMOS1713-20 | 100,00 | 24A74FS2 | Jul | 4,947330002 | -52,49211998 | 2 | P-65 |
| ***Culex vaxus*** | Cx_Zoo_0922 | FGMOS1713-20 | 100,00 | 24A74FS3 | Jul | 4,947330002 | -52,49211998 | 2 | P-65 |
| ***Culex vaxus*** | Cx_Zoo_0923 | FGMOS1713-20 | 100,00 | 24A74FS4 | Jul | 4,947330002 | -52,49211998 | 2 | P-65 |
| ***Culex vaxus*** | Cx_Zoo_0924 | FGMOS1713-20 | 100,00 | 24A74FS5 | Jul | 4,947330002 | -52,49211998 | 3 | P-65 |
| ***Culex vaxus*** | Cx_Zoo_0942 | FGMOS1713-20 | 100,00 | 26A72FS2 | Sep | 4,947476601 | -52,49250521 | 2 | P-65 |
| ***Culex vaxus*** | Cx_Zoo_0990 | FGMOS1713-20 | 100,00 | 28A80FS2 | Oct | 4,947710037 | -52,49251996 | 5 | P-65 |
| ***Culex vaxus*** | Cx_Zoo_1026 | FGMOS1713-20 | 99,85 | 31A33FS1 | Jan | 4,947610041 | -52,49244997 | 2 | P-65 |
| ***Culex vaxus*** | Cx_Zoo_1091 | FGMOS1713-20 | 100,00 | 31A29FS2 | Jan | 4,947710037 | -52,49251996 | 2 | P-65 |
| ***Culex vaxus*** | Cx_Zoo_1093 | FGMOS1713-20 | 99,85 | 31A29FS4 | Jan | 4,947710037 | -52,49251996 | 5 | P-65 |
| ***Culex vaxus*** | Cx_Zoo_1094 | FGMOS1713-20 | 100,00 | 31A29FS5 | Jan | 4,947710037 | -52,49251996 | 6 | P-65 |
| ***Culex vaxus*** | Cx_Zoo_0095 | FGMOS1713-20 | 99,85 | 04A18FS4 | Mar | 4,95107797 | -52,49196198 | 4 | P-66 |
| ***Culex vaxus*** | Cx_Zoo_0101 | FGMOS1713-20 | 100,00 | 04A18FS10 | Mar | 4,95107797 | -52,49196198 | 5 | P-66 |
| ***Culex vaxus*** | Cx_Zoo_0110 | FGMOS1713-20 | 100,00 | 05A01FS1 | Mar | 4,948890042 | -52,49287996 | 4 | P-66 |
| ***Culex vaxus*** | Cx_Zoo_1098 | FGMOS1713-20 | 100,00 | 32A91FS2 | Jan | 4,947610041 | -52,49244997 | 5 | P-66 |
| ***Culex vaxus*** | Cx_Zoo_1196 | FGMOS1713-20 | 100,00 | T2F11FS2 | Jun | 4,947819924 | -52,49239055 | 2 | P-66 |
| ***Culex vaxus*** | Cx_Zoo_0223 | FGMOS1713-20 | 100,00 | 11A12FS1 | Avr | 4,949439978 | -52,49234 | 2 | P-67 |
| ***Culex vaxus*** | Cx_Zoo_0225 | FGMOS1713-20 | 100,00 | 11A12FS3 | Avr | 4,949439978 | -52,49234 | 3 | P-67 |
| ***Culex vaxus*** | Cx_Zoo_0227 | FGMOS1713-20 | 99,85 | 11A12FS5 | Avr | 4,949439978 | -52,49234 | 5 | P-67 |
| ***Culex vaxus*** | Cx_Zoo_0329 | FGMOS1713-20 | 100,00 | 14A49FS1 | May | 4,949439978 | -52,49234 | 2 | P-67 |
| ***Culex vaxus*** | Cx_Zoo_0332 | FGMOS1713-20 | 100,00 | 14A49FS4 | May | 4,949439978 | -52,49234 | 5 | P-67 |
| ***Culex vaxus*** | Cx_Zoo_0355 | FGMOS1713-20 | 100,00 | 14A29FS1 | May | 4,948890042 | -52,49287996 | 5 | P-67 |
| ***Culex vaxus*** | Cx_Zoo_0357 | FGMOS1713-20 | 100,00 | 14A11FS2 | May | 4,947950011 | -52,49257998 | 2 | P-67 |
| ***Culex vaxus*** | Cx_Zoo_0370 | FGMOS1713-20 | 100,00 | 15A98FS1 | May | 4,947950011 | -52,49257998 | 5 | P-67 |
| ***Culex vaxus*** | Cx_Zoo_0376 | FGMOS1713-20 | 100,00 | 16A53FS1 | May | 4,949439978 | -52,49234 | 2 | P-67 |
| ***Culex vaxus*** | Cx_Zoo_0377 | FGMOS1713-20 | 100,00 | 16A53FS2 | May | 4,949439978 | -52,49234 | 4 | P-67 |
| ***Culex vaxus*** | Cx_Zoo_0378 | FGMOS1713-20 | 100,00 | 16A53FS3 | May | 4,949439978 | -52,49234 | 4 | P-68 |
| ***Culex vaxus*** | Cx_Zoo_0404 | FGMOS1713-20 | 100,00 | 17A60FS1 | Jun | 4,94896003 | -52,49238996 | 2 | P-68 |
| ***Culex vaxus*** | Cx_Zoo_0405 | FGMOS1713-20 | 100,00 | 17A60FS2 | Jun | 4,94896003 | -52,49238996 | 4 | P-68 |
| ***Culex vaxus*** | Cx_Zoo_0406 | FGMOS1713-20 | 100,00 | 17A60FS3 | Jun | 4,94896003 | -52,49238996 | 4 | P-68 |
| ***Culex vaxus*** | Cx_Zoo_0409 | FGMOS1713-20 | 99,85 | 17A29FS3 | Jun | 4,949439978 | -52,49234 | 4 | P-68 |
| ***Culex vaxus*** | Cx_Zoo_0410 | FGMOS1713-20 | 100,00 | 17A29FS4 | Jun | 4,949439978 | -52,49234 | 4 | P-68 |
| ***Culex vaxus*** | Cx_Zoo_0411 | FGMOS1713-20 | 99,85 | 17A23FS1 | Jun | 4,951195987 | -52,49194697 | 4 | P-68 |
| ***Culex vaxus*** | Cx_Zoo_0870 | FGMOS1713-20 | 100,00 | 24A08FS7 | Jul | 4,948672866 | -52,49226876 | 2 | P-68 |
| ***Culex vaxus*** | Cx_Zoo_0872 | FGMOS1713-20 | 99,85 | 24A72FS1 | Jul | 4,94896003 | -52,49238996 | 2 | P-68 |
| ***Culex vaxus*** | Cx_Zoo_1035 | FGMOS1713-20 | 100,00 | 31A87FS1 | Jan | 4,94896003 | -52,49238996 | 2 | P-68 |
| ***Culex vaxus*** | Cx_Zoo_0184 | FGMOS1713-20 | 100,00 | 10A06FS1 | Avr | 4,950990966 | -52,49061802 | 2 | P-69 |
| ***Culex vaxus*** | Cx_Zoo_0235 | FGMOS1713-20 | 100,00 | 11A62FS1 | Avr | 4,950659964 | -52,49004001 | 2 | P-69 |
| ***Culex vaxus*** | Cx_Zoo_0364 | FGMOS1713-20 | 100,00 | 15A61FS2 | May | 4,951252732 | -52,49152637 | 4 | P-69 |
| ***Culex vaxus*** | Cx_Zoo_0475 | FGMOS1713-20 | 99,85 | 20A30FS1 | Jun | 4,950801032 | -52,489751 | 2 | P-69 |
| ***Culex vaxus*** | Cx_Zoo_0547 | FGMOS1713-20 | 100,00 | 20A31FS1 | Jun | 4,950540019 | -52,49286002 | 2 | P-69 |
| ***Culex vaxus*** | Cx_Zoo_1158 | FGMOS1713-20 | 100,00 | 33A32FS1 | Jan | 4,951252732 | -52,49152637 | 3 | P-69 |
| ***Culex portesi*** | Cx_Zoo_0974 | FGMOS2416-20 | 100,00 | 27A06FS10 | Sep | 4,952010037 | -52,49096998 | 2 | P-70 |
| ***Culex portesi*** | Cx_Zoo_0982 | FGMOS2416-20 | 99,85 | 27A06FS18 | Sep | 4,952010037 | -52,49096998 | 3 | P-70 |
| ***Culex dunni*** | Cx_Zoo_0009 | FGMOS2748-20 | 97,41 | 01A18FS2 | Fev | 4,952010037 | -52,49096998 | 2 | P-71 |
| ***Culex dunni*** | Cx_Zoo_0010 | FGMOS2748-20 | 97,41 | 01A18FS3 | Fev | 4,952010037 | -52,49096998 | 2 | P-71 |
| ***Culex dunni*** | Cx_Zoo_0055 | FGMOS2750-20 | 97,72 | 02A10FS2 | Fev | 4,950780915 | -52,49287686 | n.d. | P-71 |
| ***Culex dunni*** | Cx_Zoo_0061 | FGMOS2750-20 | 97,57 | 02A10FS8 | Fev | 4,950780915 | -52,49287686 | 2 | P-71 |
