# Supplementary material for: Culex mosquitoes in a French Guiana zoo: insights on species diversity, feeding habits, and parasitic associations
Source: Parasit Vectors. 2026 May 13;19:274. doi: 10.1186/s13071-026-07377-2 (PMC13339560; doi:10.1186/s13071-026-07377-2)
Supplement: Supplementary file 5 — Additional file 5 (DOCX 27 KB) [file 13071_2026_7377_MOESM5_ESM.docx]

|  |  | **Species of mosquitoes** | | | | | | | | | | | | | | | | | | | | | | | | |  |
| --- | --- | --- | --- | --- | --- | --- | --- | --- | --- | --- | --- | --- | --- | --- | --- | --- | --- | --- | --- | --- | --- | --- | --- | --- | --- | --- | --- |
|  | **Host blood meals** | ***Culex eastor*** | ***Culex vaxus*** | ***Culex spissipes*** | ***Culex dunni*** | ***Culex pleuristriatus*** | ***Culex rabanicolus*** | ***Culex theobaldi*** | ***Culex declarator*** | ***Culex usquatus*** | ***Culex accelerans*** | ***Culex bastagarius*** | ***Culex nigripalpus*** | ***Culex phlogistus*** | ***Culex amazonensis*** | ***Culex contei*** | ***Culex portesi*** | ***Culex eknomios*** | ***Culex pedroi*** | ***Culex rabelloi*** | ***Culex tournieri*** | ***Culex innovator*** | ***Culex putumayensis*** | ***Culex lucifugus*** | ***Culex originator*** | ***Culex adamesi*** | **Total of Associations** |
| **Mammals** | *Tapirus terrestris / sp.* | **11** |  | 2 | 4 |  | 1 |  | 1 |  |  |  |  |  |  |  |  |  |  |  | 1 |  |  |  |  |  | *20* |
|  | *Dasyprocta leporina* |  | 1 | 3 | 2 |  | 1 |  | 1 |  |  |  |  |  |  |  |  |  |  |  |  |  |  |  |  | 1 | *9* |
|  | *Choloepus didactylus / sp.* |  |  | 1 | 1 | 1 | 1 | 2 | 1 |  |  |  |  |  |  |  | 1 |  |  |  |  |  |  |  |  |  | *8* |
|  | *Homo sapiens / sp.* | 1 | 3 |  |  | 1 |  | 1 |  |  |  |  |  |  |  | 1 |  |  | 1 |  |  |  |  |  |  |  | *8* |
|  | *Rattus rattus / sp.* | 1 |  |  |  |  | 2 |  |  |  | 1 |  |  |  | 1 |  | 1 |  |  |  |  |  |  |  |  |  | *6* |
|  | *Alouatta macconnelli / sp.* | 1 |  | 2 | 1 |  |  |  |  | 1 |  |  |  |  |  |  |  |  |  |  |  |  |  |  |  |  | *5* |
|  | *Cuniculus paca* | 1 |  |  | 1 |  | 1 | 1 |  |  |  |  |  |  |  |  |  |  |  |  | 1 |  |  |  |  |  | *5* |
|  | *Myrmecophaga tridactyla / sp.* | 2 |  | 1 |  |  | 1 |  |  |  | 1 |  |  |  |  |  |  |  |  |  |  |  |  |  |  |  | *5* |
|  | *Philander opossum / sp.* |  |  | 2 |  |  | 1 |  |  |  | 2 |  |  |  |  |  |  |  |  |  |  |  |  |  |  |  | *5* |
|  | *Speothos venaticus* | 2 |  | 2 |  |  |  |  | 1 |  |  |  |  |  |  |  |  |  |  |  |  |  |  |  |  |  | *5* |
|  | *Hydrochoerus hydrochaeris* |  |  |  |  | 1 |  | 1 |  |  | 1 |  |  |  |  |  |  | 1 |  |  |  |  |  |  |  |  | *4* |
|  | *Panthera onca* | 1 |  | 1 |  |  | 1 |  |  |  |  |  | 1 |  |  |  |  |  |  |  |  |  |  |  |  |  | *4* |
|  | *Tamandua tetradactyla / sp.* | 1 | 1 |  |  |  |  |  |  |  |  |  | 1 |  |  |  |  |  |  |  |  |  |  |  |  |  | *3* |
|  | *Canis lupus familiaris* | 1 |  |  |  |  |  |  | 1 |  |  |  |  |  |  |  |  |  |  |  |  |  |  |  |  |  | *2* |
|  | *Coendou prehensilis* |  |  | 1 |  |  |  |  |  | 1 |  |  |  |  |  |  |  |  |  |  |  |  |  |  |  |  | *2* |
|  | *Didelphis marsupialis / sp.* |  |  | 2 |  |  |  |  |  |  |  |  |  |  |  |  |  |  |  |  |  |  |  |  |  |  | *2* |
|  | *Eira sp.* |  |  |  |  |  |  | 1 |  |  |  |  |  |  | 1 |  |  |  |  |  |  |  |  |  |  |  | *2* |
|  | *Metachirus nudicaudatus* |  |  | 2 |  |  |  |  |  |  |  |  |  |  |  |  |  |  |  |  |  |  |  |  |  |  | *2* |
|  | *Ateles paniscus* |  |  |  |  |  |  | 1 |  |  |  |  |  |  |  |  |  |  |  |  |  |  |  |  |  |  | *1* |
|  | *Capra hircus / sp.* | 1 |  |  |  |  |  |  |  |  |  |  |  |  |  |  |  |  |  |  |  |  |  |  |  |  | *1* |
|  | *Felis sp.* | 1 |  |  |  |  |  |  |  |  |  |  |  |  |  |  |  |  |  |  |  |  |  |  |  |  | *1* |
|  | *Leopardus pardalis* | 1 |  |  |  |  |  |  |  |  |  |  |  |  |  |  |  |  |  |  |  |  |  |  |  |  | *1* |
|  | *Marmosa murina* |  |  |  |  |  |  |  |  |  |  |  |  |  |  |  |  |  |  |  |  | 1 |  |  |  |  | *1* |
|  | *Monodelphis sp.* |  |  |  |  |  |  |  |  |  |  |  |  |  |  |  |  |  |  |  |  | 1 |  |  |  |  | *1* |
|  | *Myotis sp.* | 1 |  |  |  |  |  |  |  |  |  |  |  |  |  |  |  |  |  |  |  |  |  |  |  |  | *1* |
|  | *Pecari tajacu / sp.* |  |  |  | 1 |  |  |  |  |  |  |  |  |  |  |  |  |  |  |  |  |  |  |  |  |  | *1* |
|  | *Pithecia pithecia / sp.* |  |  |  |  |  |  | 1 |  |  |  |  |  |  |  |  |  |  |  |  |  |  |  |  |  |  | *1* |
|  | *Puma yagouaroundi* |  |  |  |  |  |  |  |  |  |  |  |  |  |  |  | 1 |  |  |  |  |  |  |  |  |  | *1* |
| **Birds** | *Crax alector / sp.* | 4 | 2 | 2 |  |  | 1 |  | 1 |  |  |  | 2 |  |  |  |  |  |  |  |  |  |  |  |  |  | *12* |
|  | *Gallus gallus / sp.* | 2 |  |  | 1 |  |  |  |  |  |  |  |  |  |  |  | 1 |  |  |  |  |  |  |  |  |  | *4* |
|  | *Thamnophilus amazonicus / sp.* | 1 | 1 | 1 |  |  |  |  |  |  |  |  |  |  |  |  |  |  |  | 1 |  |  |  |  |  |  | *4* |
|  | *Turdus leucomelas / sp.* | 1 | 1 |  |  |  |  |  |  | 1 |  |  |  |  |  |  |  |  | 1 |  |  |  |  |  |  |  | *4* |
|  | *Butorides striata* | 1 | 1 |  |  |  |  | 1 |  |  |  |  |  |  |  |  |  |  |  |  |  |  |  |  |  |  | *3* |
|  | *Ramphastos toco / sp.* |  | 1 |  |  |  |  |  |  | 1 |  |  |  |  |  |  |  | 1 |  |  |  |  |  |  |  |  | *3* |
|  | *Butorides virescens* | 1 |  |  |  |  |  | 1 |  |  |  |  |  |  |  |  |  |  |  |  |  |  |  |  |  |  | *2* |
|  | *Meleagris gallopavo* |  | 1 |  |  |  | 1 |  |  |  |  |  |  |  |  |  |  |  |  |  |  |  |  |  |  |  | *2* |
|  | *Mesembrinibis cayennensis / sp.* | 1 |  | 1 |  |  |  |  |  |  |  |  |  |  |  |  |  |  |  |  |  |  |  |  |  |  | *2* |
|  | *Anas sp.* |  |  |  |  |  |  |  |  |  |  |  | 1 |  |  |  |  |  |  |  |  |  |  |  |  |  | *1* |
|  | *Buteogallus meridionalis / sp.* |  |  |  |  |  |  |  |  |  |  | 1 |  |  |  |  |  |  |  |  |  |  |  |  |  |  | *1* |
|  | *Coturnix sp.* |  |  |  | 1 |  |  |  |  |  |  |  |  |  |  |  |  |  |  |  |  |  |  |  |  |  | *1* |
|  | *Eurypyga helias* | 1 |  |  |  |  |  |  |  |  |  |  |  |  |  |  |  |  |  |  |  |  |  |  |  |  | *1* |
|  | *Myrmophylax atrothorax / sp.* |  |  | 1 |  |  |  |  |  |  |  |  |  |  |  |  |  |  |  |  |  |  |  |  |  |  | *1* |
|  | *Percnostola rufifrons* |  |  | 1 |  |  |  |  |  |  |  |  |  |  |  |  |  |  |  |  |  |  |  |  |  |  | *1* |
|  | *Ramphastos tucanus* |  | 1 |  |  |  |  |  |  |  |  |  |  |  |  |  |  |  |  |  |  |  |  |  |  |  | *1* |
|  | *Ramphastos vitellinus* |  | 1 |  |  |  |  |  |  |  |  |  |  |  |  |  |  |  |  |  |  |  |  |  |  |  | *1* |
|  | *Thamnophilus doliatus* |  | 1 |  |  |  |  |  |  |  |  |  |  |  |  |  |  |  |  |  |  |  |  |  |  |  | *1* |
|  | *Thamnophilus nigrocinereus* |  |  | 1 |  |  |  |  |  |  |  |  |  |  |  |  |  |  |  |  |  |  |  |  |  |  | *1* |
|  | *Thamnophilus punctatus* |  |  |  |  |  |  |  |  |  |  |  |  |  |  |  |  |  |  | 1 |  |  |  |  |  |  | *1* |
|  | *Thryothorus sp.* | 1 |  |  |  |  |  |  |  |  |  |  |  |  |  |  |  |  |  |  |  |  |  |  |  |  | *1* |
|  | *Turdus fumigatus* |  |  |  |  |  |  |  |  | 1 |  |  |  |  |  |  |  |  |  |  |  |  |  |  |  |  | *1* |
| **Squamates** | *Iguana iguana / sp.* | 1 | **11** | 1 | 1 | 1 |  |  |  |  |  |  |  |  |  | 1 |  | 1 | 1 |  |  |  |  |  |  |  | *18* |
|  | *Epicrates maurus / sp.* |  | 2 |  |  | 2 |  |  | 1 |  |  |  |  | 2 | 1 |  |  |  |  |  |  |  |  |  |  |  | *8* |
|  | *Eunectes murinus / sp.* |  |  |  |  |  |  |  |  |  |  |  |  | 1 |  |  |  |  |  |  |  |  |  |  |  |  | *1* |
|  | *Kentropyx sp.* | 1 |  |  |  |  |  |  |  |  |  |  |  |  |  |  |  |  |  |  |  |  |  |  |  |  | *1* |
|  | *Leptophis ahaetulla / sp.* |  |  |  |  |  |  |  |  |  |  |  |  |  |  |  |  |  |  |  |  |  |  | 1 |  |  | *1* |
| **Amphibians** | *Osteocephalus taurinus / sp.* |  |  |  | 2 | 1 |  |  | 1 | 1 |  |  |  | 2 |  |  |  |  |  | 1 |  |  | 1 |  | 1 |  | *10* |
|  | *Scinax ruber / sp.* |  | 2 |  |  | 3 |  |  |  |  |  |  |  |  |  | 1 |  |  |  |  |  |  | 1 |  |  |  | *7* |
|  | *Rhinella marina / sp.* |  |  |  |  |  |  |  |  |  |  | 2 |  |  | 1 |  |  |  |  |  |  |  |  |  |  |  | *3* |
|  | *Leptodactylus pentadactylus / sp.* |  |  |  |  |  |  |  |  |  |  | 1 |  |  |  |  |  |  |  |  |  |  |  |  |  |  | *1* |
|  | *Trachycephalus typhonius / sp.* |  |  |  |  | 1 |  |  |  |  |  |  |  |  |  |  |  |  |  |  |  |  |  |  |  |  | *1* |
| **Crocodilia** | *Caiman crocodilus / sp.* | 1 | 2 | 1 |  |  |  |  |  |  |  |  |  |  |  |  |  |  |  |  |  |  |  |  |  |  | *4* |
|  | *Paleosuchus palpebrosus / sp.* | 1 | 1 |  |  |  |  |  |  |  |  | 1 |  |  |  | 1 |  |  |  |  |  |  |  |  |  |  | *4* |
|  | *Melanosuchus niger* | 1 | 2 |  |  |  |  |  |  |  |  |  |  |  |  |  |  |  |  |  |  |  |  |  |  |  | *3* |
| **Testudines** | *Chelonoidis carbonarius / sp.* | 1 |  |  |  |  |  |  |  |  |  |  |  |  |  |  |  |  |  |  | 1 |  |  |  |  |  | *2* |
|  | *Chelonoidis denticulatus* | 1 |  |  |  |  |  |  |  |  |  |  |  |  |  |  |  |  |  |  |  |  |  |  |  |  | *1* |
|  | ***Total*** | ***46*** | ***35*** | ***28*** | ***15*** | ***11*** | ***11*** | ***10*** | ***8*** | ***6*** | ***5*** | ***5*** | ***5*** | ***5*** | ***4*** | ***4*** | ***4*** | ***3*** | ***3*** | ***3*** | ***3*** | ***2*** | ***2*** | ***1*** | ***1*** | ***1*** | ***221*** |
