# Supplementary material for: Culex mosquitoes in a French Guiana zoo: insights on species diversity, feeding habits, and parasitic associations
Source: Parasit Vectors. 2026 May 13;19:274. doi: 10.1186/s13071-026-07377-2 (PMC13339560; doi:10.1186/s13071-026-07377-2)
Supplement: Supplementary file 6 — Additional file 6 (PDF 43 KB) [file 13071_2026_7377_MOESM6_ESM.pdf]

Mosquitoes species (DNA barcode)	ID Positive Pool	Samples nb	Mammals	Birds	Crocodilians	Squamates	Turtles	Amphibians	Haemoparasites / GenBank Accession (% ident.)	Trypanosomes / GenBank Accession (% ident.)
Culex bastagarius	P-04	10	*n.d.*	*Buteogallus meridionalis/sp. (Accipitriformes)*	*Paleosuchus palpebrosus/sp. (Crocodilia)*	*n.d.*	*n.d.*	*Rhinella marina/sp.(Amphibians)*	/	Trypanosoma sp. 858 / EU021228 (99,60%)
	P-05	4	*n.d.*	*n.d.*	*n.d.*	*n.d.*	*n.d.*	*Rhinella marina/sp.(Amphibians), Leptodactylus pentadactylus/sp. (Amphibians)*	/	Trypanosoma sp. 858 / EU021228 (100,00%)
Culex contei	P-06	9	*n.d.*	*n.d.*	*Paleosuchus palpebrosus/sp. (Crocodilia)*	*Iguana iguana (lizards)*	*n.d.*	*n.d.*	/	Trypanosoma sp. CMP-2015 / KM406915 (92,56%)
	P-07	9	*Homo sapiens/sp. (Human)*	*n.d.*	*n.d.*	*n.d.*	*n.d.*	*Scinax ruber/sp. (Amphibians)*	/	Trypanosoma sp. 362 / EU021232 (99,81%)
Culex declarator	P-10	8	*Canis lupus familiaris (Carnivora), Choloepus didactylus/sp. (Pilosa), Dasyprocta leporina (Rodentia), Speothos venaticus (Carnivora)*	*Crax alector/sp. (Galliformes)*	*n.d.*	*Epicrates maurus/sp. (Snakes)*	*n.d.*	*n.d.*	/	Endotrypanum sp. 889 / EU021240 (99,93%)
Culex dunni	P-12	9	*Alouatta sp. (Non-human primates)*	*n.d.*	*n.d.*	*Iguana iguana (lizards)*	*n.d.*	*n.d.*	Plasmodium floridense / NC_009961 (96,90%)	Paratrypanosoma sp. M2031 / OP748979 (98,49%)
Culex eastor	P-09	1	*Myotis sp. (Chiroptera)*	*Thryothorus sp. (Passeriformes)*	*n.d.*	*Kentropyx sp. (lizards)*	*n.d.*	*n.d.*	Co-occurence : Plasmodium kentropyxi / KY653753 (97,08%) et Plasmodium carmelinoi / MF177708 (97,86%)	/
	P-18	8	*Tapirus terrestris/sp. (Perissodactyla), Speothos venaticus (Carnivora), Leopardus pardalis (Carnivora)*	*Turdus leucomelas (Passeriformes), Crax alector (Galliformes)*	*n.d.*	*n.d.*	*n.d.*	*n.d.*	Plasmodium sp. HMA-2012 CRGEN3 / JN819341 (100,00%)	/
	P-19	10	*Tapirus terrestris/sp. (Perissodactyla), Myrmecophaga tridactyla/sp. (Pilosa)*	*Gallus gallus (Galliformes), Butorides striata (Pelecaniformes), Butorides virescens (Pelecaniformes)*	*n.d.*	*n.d.*	*n.d.*	*n.d.*		Trypanosoma avium / KT728402 (98,57%)
	P-20	10	*Tapirus terrestris/sp. (Perissodactyla), Tamandua tetradactyla (Pilosa)*	*n.d.*	*n.d.*	*n.d.*	*n.d.*	*n.d.*	/	Trypanosoma minasense / PQ490334 (98,11%)
	P-21	10	*Canis lupus familiaris (Carnivora), Tapirus terrestris/sp. (Perissodactyla)*	*Gallus gallus/sp. (Galliformes), Eurypyga helias (Eurypygiformes),*	*n.d.*	*n.d.*	*n.d.*	*n.d.*	/	Paratrypanosoma sp. M2031 / OP748979 (98,47%)
	P-23	10	*Tapirus terrestris/sp. (Perissodactyla), Alouatta sp. (Non-human primates), Panthera onca (Carnivora), Speothos venaticus (Carnivora)*	*n.d.*	*n.d.*	*n.d.*	*n.d.*	*n.d.*	Plasmodium floridense / NC_009961 (96,77%)	/
	P-24	8	*Capra hircus/sp. (Artiodactyla),(Perissodactyla)*	*Crax alector (Galliformes)*	*Melanosuchus niger (Crocodilia)*	*n.d.*	*Chelonoidis denticulatus (Turtles), Chelonoidis sp. (Turtles)*	*n.d.*	Plasmodium kentropyxi / KY653753 (98,40%)	/
	P-27	10	*Myrmecophaga tridactyla (Pilosa), Tapirus terrestris/sp. (Perissodactyla)*	*n.d.*	*n.d.*	*n.d.*	*n.d.*	*n.d.*	/	Trypanosoma terrestris / KF586848 (99,67%)
Culex eknomios	P-29	4	*Hydrochoerus hydrochaeris (Rodentia)*	*Ramphastos toco (Piciformes)*	*n.d.*	*Iguana iguana/sp. (lizards)*	*n.d.*	*n.d.*	/	Trypanosoma culicavium / HQ107967 (98,19%)

Culex innovator	P-31	4	*Marmosa murina (Didelphimorphia), Monodelphis sp. (Didelphimorphia)*	*n.d.*	*n.d.*	*n.d.*	*n.d.*	*n.d.*	/	Trypanosoma sp. CMP-2015 / KM406915 (92,56%)
Culex phlogistus	P-38	6	*n.d.*	*n.d.*	*n.d.*	*Eunectes murinus/sp. (Snakes), Epicrates maurus/sp. (Snakes)*	*n.d.*	*n.d.*	Co-occurence : Plasmodium kentropyxi / KY653753 (97,08%) et Plasmodium carmelinoi / MF177708 (97,86%)	Trypanosomatidae sp. DN359 / MW869896 (98,85%)
	P-39	4	*n.d.*	*n.d.*	*n.d.*	*n.d.*	*n.d.*	*Osteocephalus taurinus (Amphibians)*	/	Trypanosoma sp. 362 / EU021232 (97,38%)
	P-08	8	*n.d.*	*n.d.*	*n.d.*	*Epicrates maurus/sp. (Snakes)*	*n.d.*	*Osteocephalus taurinus (Amphibians)*	/	Trypanosoma sp. 362 / EU021232 (99,61%)
Culex pleuristriatus	P-41	7	*Homo sapiens (Human)*	*n.d.*	*n.d.*	*n.d.*	*n.d.*	*Scinax ruber (Amphibians)*	/	Trypanosoma sp. 444 / EU021225 (92,97%)
	P-42	10	*Hydrochoerus hydrochaeris (Rodentia)*	*n.d.*	*n.d.*	*Epicrates sp. (Snakes), Iguana iguana/sp. (lizards)*	*n.d.*	*Scinax ruber (Amphibians)*	Plasmodium floridense / NC_009961 (96,77%)	Trypanosoma sp. 406 / EU021236 (97,25%)
Culex rabanicolus	P-47	9	*Cuniculus paca (Rodentia), Rattus rattus (Rodentia), Philander opossum/sp. (Didelphimorphia)*	*n.d.*	*n.d.*	*n.d.*	*n.d.*	*n.d.*	/	Trypanosomatidae sp. M2030 / OP748978 (97,96%)
	P-48	10	*Tapirus terrestris (Perissodactyla), Choloepus didactylus/sp. (Pilosa), Dasyprocta leporina (Rodentia), Rattus rattus (Rodentia), Myrmecophaga tridactyla (Pilosa)*	*n.d.*	*n.d.*	*n.d.*	*n.d.*	*n.d.*	/	Trypanosomatidae sp. M2030 / OP748978 (97,25%)
Culex rabelloi	P-49	10	*n.d.*	*Thamnophilus punctatus/amazonicus/sp. (Passeriformes)*	*n.d.*	*n.d.*	*n.d.*	*Osteocephalus taurinus (Amphibians)*	/	Co-occurence : Trypanosoma sp. N335 / AJ223570 (98,77%) and Trypanosoma sp. 362 / EU021232 (99,61%)
Culex spissipes	P-50	6	*Alouatta macconnelli/sp. (Non-human primates),Dasyprocta leporina (Rodentia), Speothos venaticus (Carnivora)*	*Crax alector (Galliformes)*	*n.d.*	*Iguana iguana (lizards)*	*n.d.*	*n.d.*	Plasmodium floridense / NC_009961 (96,77%)	Trypanosomatidae sp. M2030 / OP748978 (98,60%)
	P-53	10	*Tapirus terrestris (Perissodactyla), Philander opossum/sp. (Didelphimorphia), Choloepus didactylus (Pilosa), Alouatta macconnelli/sp. (Non-human primates)*	*n.d.*	*n.d.*	*n.d.*	*n.d.*	*n.d.*	Plasmodium nucleophilum / OP701684 (99,74%)	Trypanosomatidae sp. M2030 / OP748978 (97,66%)
	P-54	6	*Tapirus terrestris/sp. (Perissodactyla), Dasyprocta leporina (Rodentia), Coendou prehensilis (Rodentia), Myrmecophaga tridactyla (Pilosa)*	*n.d.*	*Caiman crocodilus/sp. (Crocodilia)*	*n.d.*	*n.d.*	*n.d.*	Plasmodium sp. HMA-2012 BTFG1 / JN819338 (98,01%)	/
Culex theobaldi	P-56	7	*Pithecia pithecia/sp. (Non-human primates), Eira sp. (Carnivora), Homo sapiens (Human) , Ateles paniscus (Non-human primates), Choloepus didactylus (Pilosa)*	*Butorides virescens (Pelecaniformes), Butorides striata (Pelecaniformes)*	*n.d.*	*n.d.*	*n.d.*	*n.d.*	/	Trypanosoma culicavium / HQ909083 (96,84%)

Culex usquatus	P-58	5	*Coendou prehensilis (Rodentia), Alouatta sp. (Non-human primates)*	*Turdus leucomelas/funigatus/sp. (Passeriformes), Ramphastos toco (Piciformes)*	*n.d.*	*n.d.*	*n.d.*	*Osteocephalus taurinus (Amphibians)*	Plasmodium tejerai / JX467689 (99,48%)	Trypanosoma sp. 362 / EU021232 (98,95%)
	P-63	10	*Homo sapiens (Human)*	*n.d.*	*n.d.*	*Iguana iguana/sp. (lizards)*	*n.d.*	*n.d.*	Plasmodium floridense / NC_009961 (96,90%)	Trypanosomatidae sp. M2030 / OP748978 (98,37%)
	P-64	10	*n.d.*	*n.d.*	*Caiman crocodilus/sp. (Crocodilia)*	*Iguana iguana (lizards)*	*n.d.*	*n.d.*	Plasmodium floridense / NC_009961 (96,90%)	Paratrypanosoma sp. M2031 / OP748979 (97,49%)
Culex vaxus	P-65	10	*Homo sapiens (Human)*	*n.d.*	*n.d.*	*Iguana iguana/sp. (lizards)*	*n.d.*	*n.d.*	Plasmodium floridense / NC_009961 (96,90%)	Co-occurence : Paratrypanosoma sp. M2031 / OP748979 (98,41%), Neobodo curvifilus / AY425015 (96,73%) and Parabodo caudatus / DQ207591 (97,44%)
	P-67	10	*n.d.*	*Ramphastos tucanus/vitellinus/sp. (Piciformes)*	*n.d.*	*Iguana iguana/sp. (lizards)*	*n.d.*	*Scinax sp. (Amphibians)*	Plasmodium floridense / NC_009961 (96,90%)	Trypanosomatidae sp. M2030 / OP748978 (97,93%)
