# Supplementary material for: Culex mosquitoes in a French Guiana zoo: insights on species diversity, feeding habits, and parasitic associations
Source: Parasit Vectors. 2026 May 13;19:274. doi: 10.1186/s13071-026-07377-2 (PMC13339560; doi:10.1186/s13071-026-07377-2)
Supplement: Supplementary file 7 — Additional file 7 (DOCX 16 KB) [file 13071_2026_7377_MOESM7_ESM.docx]

| **Mosquitoes species** | **Nb of pool** | **Total samples in pools** | **Number of host species of blood-fed females** | | | | | | | **Positives Plasmodium/ Haemoparasites pools (% rate of infection)** | **Positives trypanosomes pools (% rate of infection)** |
| --- | --- | --- | --- | --- | --- | --- | --- | --- | --- | --- | --- |
|  |  |  | **MAMMALS** | **SAUROPSIDS** | | | | **AMPHIBIANS** | **Human pools (HBI %)** |  |  |
|  |  |  |  | **BIRDS** | **CROCODILIANS** | **SQUAMATES** | **TURTLES** |  |  |  |  |
| ***Culex accelerans*** | 1 | 4 | 4 | - | - | - | - | - | - | - | - |
| ***Culex adamesi*** | 1 | 1 | 1 | - | - | - | - | - | - | - | - |
| ***Culex amazonensis*** | 1 | 8 | 2 | - | - | 1 | - | 1 | - | - | - |
| ***Culex bastagarius*** | 2 | 14 | - | 1 | 1 | - | - | 2 | - | - | **2 (14.3)** |
| ***Culex contei*** | 2 | 18 | 1 | - | 1 | 1 | - | 1 | **1 (5.6)** | - | **3 (16.7)** |
| ***Culex declarator*** | 2 | 13 | 5 | 1 | - | 1 | - | 1 | - | - | **1 (7.7)** |
| ***Culex dunni*** | 5 | 39 | 6 | 2 | - | 1 | - | 1 | - | **1 (2.6)** | **1 (2.6)** |
| ***Culex eastor*** | 14 | 113 | 12 | 8 | 3 | 2 | 2 | - | **1 (0.9)** | **4 (3.5)** | **4 (3.5)** |
| ***Culex eknomios*** | 1 | 4 | 1 | 1 | - | 1 | - | - | - | - | **1 (25.0)** |
| ***Culex innovator*** | 1 | 4 | 2 | - | - | - | - | - | **1 (25.0)** | - | **2 (50.0)** |
| ***Culex lucifugus*** | 1 | 1 | - | - | - | 1 | - | - | - | - | - |
| ***Culex nigripalpus*** | 2 | 10 | 2 | 2 | - | - | - | - | - | - | - |
| ***Culex originator*** | 1 | 1 | - | - | - | - | - | 1 | - | - | - |
| ***Culex pedroi*** | 1 | 5 | 1 | 1 | - | 1 | - | - | **1 (20.0)** | - | - |
| ***Culex phlogistus*** | 3 | 18 | - | - | - | 2 | - | 1 | - | **2 (11.1)** | **3 (16.7)** |
| ***Culex pleuristriatus*** | 4 | 36 | 3 | - | - | 2 | - | 3 | **1 (2.8)** | **1 (2.8)** | **1 (2.8)** |
| ***Culex portesi*** | 1 | 7 | 3 | 1 | - | - | - | - | - | - | - |
| ***Culex putumayensis*** | 1 | 10 | - | - | - | - | - | 2 | - | - | - |
| ***Culex rabanicolus*** | 3 | 21 | 9 | 2 | - | - | - | - | - | - | **1 (4.8)** |
| ***Culex rabelloi*** | 1 | 10 | - | 2 | - | - | - | 1 | - | - | **1 (10.0)** |
| ***Culex spissipes*** | 5 | 39 | 11 | 5 | 1 | 1 | - | - | - | **3 (7.7)** | **1 (2.6)** |
| ***Culex theobaldi*** | 2 | 16 | 7 | 2 | - | - | - | - | **1 (6.3)** | - | **1 (6.3)** |
| ***Culex tournieri*** | 1 | 6 | 2 | - | - | - | 1 | - | - | - | - |
| ***Culex usquatus*** | 1 | 5 | 2 | 3 | - | - | - | 1 | - | **1 (20.0)** | **1 (20.0)** |
| ***Culex vaxus*** | 11 | 101 | 3 | 7 | 3 | 2 | - | 1 | **3 (3.0)** | **4 (4.0)** | **6 (5.9)** |
| ***Total*** | *68* | *504* |  |  |  |  |  |  |  | *17* | *29* |
